# Melatonin: Regulation of Viral Phase Separation and Epitranscriptomics in Post-Acute Sequelae of COVID-19

**DOI:** 10.3390/ijms23158122

**Published:** 2022-07-23

**Authors:** Doris Loh, Russel J. Reiter

**Affiliations:** 1 Independent Researcher, Marble Falls, TX 78654, USA; lohdoris23@gmail.com; 2 Department of Cell Systems and Anatomy, UT Health San Antonio, San Antonio, TX 78229, USA

**Keywords:** melatonin, liquid-liquid phase separation, depolarization, nucleocapsid, m^6^A, LINE1, DDX3X, GSK-3, G3BP1, stress granule

## Abstract

The relentless, protracted evolution of the SARS-CoV-2 virus imposes tremendous pressure on herd immunity and demands versatile adaptations by the human host genome to counter transcriptomic and epitranscriptomic alterations associated with a wide range of short- and long-term manifestations during acute infection and post-acute recovery, respectively. To promote viral replication during active infection and viral persistence, the SARS-CoV-2 envelope protein regulates host cell microenvironment including pH and ion concentrations to maintain a high oxidative environment that supports template switching, causing extensive mitochondrial damage and activation of pro-inflammatory cytokine signaling cascades. Oxidative stress and mitochondrial distress induce dynamic changes to both the host and viral RNA m^6^A methylome, and can trigger the derepression of long interspersed nuclear element 1 (LINE1), resulting in global hypomethylation, epigenetic changes, and genomic instability. The timely application of melatonin during early infection enhances host innate antiviral immune responses by preventing the formation of “viral factories” by nucleocapsid liquid-liquid phase separation that effectively blockades viral genome transcription and packaging, the disassembly of stress granules, and the sequestration of DEAD-box RNA helicases, including DDX3X, vital to immune signaling. Melatonin prevents membrane depolarization and protects cristae morphology to suppress glycolysis via antioxidant-dependent and -independent mechanisms. By restraining the derepression of LINE1 via multifaceted strategies, and maintaining the balance in m^6^A RNA modifications, melatonin could be the quintessential ancient molecule that significantly influences the outcome of the constant struggle between virus and host to gain transcriptomic and epitranscriptomic dominance over the host genome during acute infection and PASC.

## 1. Introduction

The severe acute respiratory syndrome coronavirus 2 (SARS-CoV-2) pandemic has infected more than 528 million individuals and claimed the lives of over 6 million around the world since January 2020. The successful implementation of global immunization programs with ~8.5 billion doses delivered as of late 2021 may have slowed the rate of hospitalization and mortality. However, the successful immune escape by SARS-CoV-2 has resulted in the continuous emergence of potent variants such as Omicron and sub-variants that can evade neutralization by vaccinated sera [1]. These mutations typically emerge at antigenic sites that are intrinsically disordered with high structural flexibility critical for antibody escape and immune evasion [2]. Although breakthrough infections from declining antibody response post-vaccination as well as variant resistance may account for persistent symptoms in ~19% of fully vaccinated individuals [3,4], ~85.9% of COVID-19 patients from an international cohort of 3762 participants from 56 countries over a period of seven months beyond acute infection reported significant disability from 203 residual symptoms involving 10 organ systems [5]. A scoping review that analyzed 50 reports that included cohort and cross-sectional studies conducted in Europe (37 studies), Asia (6), North America (4), South America (1), Africa (1), and an unclarified location discovered more than 100 persistent symptoms reported by participants at ≥ 4 weeks post-infection [6].

The post-acute sequelae of COVID-19 (PASC) may include protracted symptoms of fatigue, dyspnea, cough, chest tightness, joint stiffness, olfactory dysfunction, and headache [7], while pulmonary, cardiovascular, neuropsychiatric, and gastrointestinal syndromes remain as dominant manifestations of PASC [8]. Most “long-haulers’’ may experience relapses triggered by physical or mental stress, whereas cognitive dysfunction or memory issues are common regardless of age [5]. Due to the potential severity and lack of viable treatments, the National Institute of Health (NIH) launched an initiative with USD 1.15 billion in grant funding to investigate and find treatment solutions for PASC [9]. The presence of a viral reservoir in PASC patients may explain improvement of clinical symptoms upon administration of SARS-CoV-2 vaccines [10]. Viral reservoirs are cells or anatomical sites where a replication-competent form of the virus can persist and accumulate with higher kinetic stability than the main pool of actively replicating viruses [11]. The prolonged presence of replication-competent SARS-CoV-2 viral ribonucleic acid (RNA) in mildly symptomatic or clinically recovered individuals is extensively documented [12,13,14]. Even in asymptomatic individuals, 50% of intestinal biopsies obtained at 4 months after COVID-19 infection displayed a lingering presence of SARS-CoV-2 nucleic acids and immunoreactivity [15]. 

The prolonged and persistent symptoms in PASC are increasingly associated with the presence of viral RNA in potential SARS-CoV-2 reservoirs located in extrapulmonary organs and tissues [16,17], including the central nervous system (CNS) [18]; ocular surface tissues [19,20], ocular fluids [21,22], and retinal/photoreceptor cells [23]; the olfactory epithelium [24]; the gastrointestinal tract [25] and feces [12,26]; injured skin [27]; as well as adipose tissues [28]. In the pharyngeal mucosa [29] and the oral mucosa [30], salivary glands can be reservoirs for productive replication and transmission [31], while periodontal pockets may act as viable anatomical environments for rapid viral dissemination and infection of distant extrapulmonary organs and tissues via gingival peripheral blood vessel interactions with the circulatory system [17,32,33]. 

## 2. Viral Persistence May Modulate Innate Immune Response

Many non-retroviral, single-stranded RNA viruses, including the Ebola virus [34], measles virus [35,36], Zika virus [37], and SARS-CoV-2 [38,39], can establish viral reservoirs within a population, employing different mechanisms to increase viral persistence in hosts that can lead to chronic disease or relapses of acute infection [40]. The Ebola virus is a negative, single-stranded RNA virus [41] capable of extended viral persistence in semen [42]. Genomic samples from patients in Guinea infected by Zaire ebolavirus in 2021 revealed a clear lower divergence, supporting the theory that viral persistence and reactivation can occur on timescales five years or longer to cause a fresh outbreak seven years after the first epidemic [43]. The Zika virus (ZIKV) can also persist in semen three months or longer after symptom onset [44], while its persistence in placental tissues can continue to infect and replicate in fetal brains for several months after initial maternal infection to cause potential long-term neurocognitive deficits after birth [45,46,47].

The ZIKV is a single-stranded RNA virus belonging to the family *Flaviviridae*, genus *Flavivirus* [48]. In 1986, Kristensson and Norrby identified seven families of single-stranded RNA viruses—*Picornaviridae*, *Togaviridae*, *Coronaviridae*, *Arenaviridae*, *Rhabdoviridae*, *Paramyxoviridae*, and *Retroviridae*—that are capable of establishing persistent infections in the CNS but excluded *Flaviviridae* due to limited data available at the time [49]. Recent work with non-lethal neonatal ZIKV mouse models found the presence of ZIKV in the CNS of acute infection survivors after more than one year to not only interfere with healing but also contribute to the progressive development of cognitive impairment and behavioral deficit. The extended presence of ZIKV may also explain the continued increase in the expression of inflammatory genes and pro-inflammatory cytokines such as interferon-gamma (IFN-γ) in the CNS more than one year post-infection [37].

Similarly, in a large, longitudinal cohort of 1096 patients infected by the SARS-CoV-2 virus with mild to critical initial disease, 46.9% reported common symptoms such as fatigue, sleep irregularities, and muscle weakness 12 months post-infection. Even though 16 of these patients tested negative for neutralizing antibodies after 12 months, 94% (15/16) exhibited SARS-CoV-2 T-cell immune response including IFN-γ [50], matching the results from another study examining mild COVID-19 and the persistence of symptoms and immune response 12 months post-infection, where two-thirds of the patients presented specific IFNγ-producing T-cells [51]. Furthermore, there is evidence of antigenic persistence where the continued memory B cells clonal turnover is observed in individuals even at 6.2 months after recovery from COVID-19 infection. This active humoral response may express antibodies characterized by enhanced somatic hypermutation, potency, and resistance to mutations in the SARS-CoV-2 receptor-binding domain (RBD) [15]. However, viral persistence in infected patients with positive COVID-19 retest results was associated with the failure to create a robust protective humoral immune response [25] that ultimately contributes to successful immune escape and emergence of new variants which can further perpetrate viral persistence in individuals with intact immune responses [52,53]. In fact, the hallmark of COVID-19 disease pathology and progression is the deficiency of antiviral interferon (IFN) responses that restrict viral production and promote viral clearance. The global interference with the expression and production of host genes resulting in the effective antagonism and suppression of the IFN signaling pathway are mediated by diverse strategies employed by SARS-CoV-2 during infection and replication [54,55,56]. First and foremost is the formation of viral condensates via liquid-liquid phase separation that facilitates viral transcription and genome packaging to support replication and dissemination.

## 3. SARS-CoV-2 Proteins Phase Separation Disrupt Host Biomolecular Condensates That Regulate Gene Expression and Interferon Immune Signaling

Liquid-liquid phase separation (LLPS)—a rapid, energy-efficient, thermodynamic process fueled mainly by the reduction or a negative change in global free energy—is the fundamental driving force behind the formation and dissolution of membraneless biomolecular condensates [57,58,59] in all living organisms, including eukaryotes, prokaryotes, and archaea [60,61,62,63]. Biomolecular condensates are reversible, micron-scale, membraneless, intracellular compartments that efficiently organize cellular biochemistry by concentrating and/or sequestering different proteins, RNAs, and other nucleic acids. Increasing the concentration of resident molecules can accelerate chemical reactions within the complex, whereas sequestration of molecules such as transcription factors can inhibit their reactions outside the complex [64]. Viruses often manipulate host biomolecular condensates that sequester translationally stalled messenger RNAs (mRNA) to maximize replication [65]. As a result, many viruses target stress granules (SGs), which are cytoplasmic, membraneless condensates that temporarily sequester non-translating mRNAs and RNA-binding proteins (RBPs) to stall host bulk translation and limit viral protein accumulation [66,67]. Pathogenic viral infections trigger the host integrated stress response (ISR) which immediately initiates the swift formation of SGs that act as emergency triage signaling hubs to regulate both mRNA translation and repression in order to promote cell survival [68,69,70,71,72]. Cells depend upon LLPS to support the timely and energy efficient assembly of SGs and other biomolecular condensates that can regulate immune signaling during viral infection [73].

The ISR comprises four early-responder kinases that phosphorylate eukaryotic translation initiation factor 2 alpha (eIF2⍺), which is the core of ISR [74]. Viral infections can activate one of the four ISR stress kinases—the double-stranded RNA-dependent protein kinase (PKR) which is induced by interferon [75,76,77]—leading to the formation of SGs that not only enhance antiviral innate immune signaling [78] but also inhibit viral protein accumulation and replication [66,79]. Upon viral infection, PKR is activated by autophosphorylation triggered by conformational changes upon binding to viral double-strand RNA (dsRNA) that are intermediates of viral replication [80,81]. The mammalian orthoreovirus uses its double-stranded RNA-binding protein σ3 to inhibit PKR activation and suppress SG formation, causing myocarditis in infected mice [82], while SARS-CoV-2 N protein inhibits PKR autophosphorylation and activation via an RNA-dependent interaction with PKR to suppress SG formation [80]. In addition to induction by ISR phosphorylation during viral infections, SGs are activated by various endogenous and exogenous stress signals [83,84], including oxidative stress [85,86,87], nutrient deprivation [88,89], ultraviolet irradiation [90,91], hypoxia [92,93], and endoplasmic reticulum (ER) stress [94,95,96]. Increased cellular oxidative stress resulting in formation of SGs can actually provoke the reactivation of persistent viral infections by enhancing access to viral molecular condensates that facilitate viral replication through the colocalization with SGs.

Virus nucleocapsid (N) proteins that adopt homogenous conformations due to prolonged stress have increased accessibility to viral genomes that enhance transcription and replication, and exposure to acute or prolonged mild oxidative stress can alter interactions of proteins within viral condensates to facilitate the transition from slow viral replication during persistent infections to activated viral replication that upregulated transcription and virion budding by 2- to 4-fold [97]. Thus, the necessity to modulate the formation and function of SGs is likely prioritized by viruses to ensure successful viral replication that is dependent upon the host translation system. As such, many single-stranded RNA viruses including the dengue virus [98], Japanese encephalitis virus [99], measles virus [100], West Nile virus [101], Usutu virus [102], and Zika virus [103], all evolved successful mechanisms to modulate and interfere with host SG induction and formation. The SARS-CoV-2 virus is no exception.

### 3.1. SARS-CoV-2 Evades Host Interferon Responses by Inhibition of the JAK-STAT Signaling Pathway in a Time-Sensitive Manner

The first line of defense in vertebrates against viral infection is the evolutionarily conserved innate interferon (IFN) immune system responsible for potent antiviral responses that inhibit the replication and spread of viruses in the absence of adaptive immunity [104,105]. The ISR PKR kinase is activated by IFN [75,76,77]. Infections by double-stranded, negative- and positive-strand RNA viruses, as well as DNA viruses, activate the production of IFNs that initiate a concerted antiviral signaling cascade mediated by the Janus kinase (JAK)-signal transducers and activators of transcription (STAT) signaling pathway [106,107]. IFN signaling upregulates the expression of interferon-stimulated genes (ISGs) that can confer significant viral interference to disrupt viral formation and replication [108]. Viruses have evolved highly successful mechanisms to block IFN-stimulated gene production in order to control and counteract IFN antiviral signaling [109]. The Japanese encephalitis virus (JEV) flavivirus is highly efficient in blocking the IFN-induced activation of JAK-STAT signaling cascade [110], while IFN inhibition, resistance, attenuation, and evasion by SARS-CoV-2 and variants via various mechanisms have been reported in great detail [54,111,112,113,114,115]. Deficiencies in the first line IFN defense system may result in impaired type I IFN responses resulting in high blood viral load that are often associated with severe and critical COVID-19 patients [116], while the JAK-STAT signaling pathway may also be suppressed during post-infection by persistent viral reservoirs.

During the acute infection phase, SARS-CoV-2 can inhibit signal transducer and activator of transcription 1 (STAT1), elevating a compensatory hyperactivation of STAT3 that results in hypercytokinemia [117,118,119]. Therefore, the inhibition of JAK-STAT signaling can potentially attenuate these runaway inflammatory responses [120,121]. Even though JAK-STAT chemical inhibitors such as ruxolitinib, baricitinib, and tofacitinib may be effective treatment candidates for SARS-CoV-2-associated inflammatory cytokine storm, respiratory failure, dysregulated thrombotic process, and multiorgan dysfunction [120,122,123,124,125,126], SARS-CoV-2 infection was actually enhanced via chemical inhibition of JAK kinases by ruxolitinib and baricitinib in human induced pluripotent stem cell differentiated into cardiomyocytes (hiPSC-CMs) that are susceptible to SARS-CoV-2 infection [127]. Moreover, SARS-CoV-2 has evolved sophisticated mechanisms to evade IFN signaling [128,129]. A post-infection systematic analysis across diverse cell types revealed pervasive targeting of the proximal components of the JAK-STAT signaling pathway, including Janus kinase 1 (JAK1), tyrosine kinase 2 (Tyk2), and the interferon receptor subunit 1 (IFNAR1) that resulted in cellular desensitization to type I IFN, resistance to IFN-⍺, and a universal inhibition of interferon signaling, where a 90% suppression of STAT phosphorylation was observed in SARS-CoV-2-infected cells compared to uninfected cells [127].

Despite robust induction of type I and III IFNs, primary human airway epithelia (HAE) and lung cells infected by SARS-CoV-2 were unable to suppress viral replication unless they were pretreated with exogenous type I IFN. Consequently, even IFN treatment as early as 8 h post-infection had no significant impact on the reduction of viral replication rate [130]. Similarly, baicalein—a natural bioactive phenolic flavonoid compound obtained from the root of *Scutellaria baicalensis*—which can strongly inhibit recruitment the SARS-CoV-2 RNA-dependent RNA polymerase (RdRp) through specific binding, was most effective only from 2 h pre-infection up to 10 h post-infection [131,132]. However, early induction of T lymphocytes that secrete IFNs targeting SARS-CoV-2 is associated with patients who exhibited milder symptoms and accelerated viral clearance [133]. The fact that the IFN innate immune signaling system in healthy children is primed and ready in a preactivated state across several epithelial cell types may also explain milder COVID-19 disease severity in children compared to adults as a result of increased timely responsiveness to viral attack [134,135,136,137]. In addition, type I IFNs and ISGs are poorly induced especially after the establishment of SARS-CoV-2 infection, but the blockade of IFN signaling can be largely impeded by IFN pretreatment (6 h preinfection) while post-infection treatment at 16 h yielded only modest results [138]. Higher levels of plasma melatonin in children may mediate the priming and preactivation of their IFN immune response systems. Compared to adults, children under the age of 15 have considerably higher levels of nocturnal plasma melatonin, with the highest concentrations found in children between ages one and three (329.5 ± 42.0 pg/mL), whereas children < 6 months had the lowest levels (27.3 ± 5.4 pg/mL)—not dissimilar to adults 70–90 years of age (29.2 ± 6.1 pg/mL) [139].

### 3.2. The Effects of Melatonin Preactivation of the IFN Signaling Response Are Time- and Dose-Dependent

Melatonin is extensively reviewed and documented for its potent antiviral properties [140,141,142,143,144,145] that can activate type I IFN-⍺ responsible for promoting JAK1/2 signaling and phosphorylation of STAT3 [146,147,148,149]. Leukocytes, including neutrophils, are largely responsible for the production of IFN-⍺ [150,151], and melatonin can increase the production of leukocytes. Human volunteers supplemented with 20 mg melatonin exhibited enhanced leukocyte chemokine expression and leukocyte chemotactic response, while 1 nM physiological concentration of melatonin via intraperitoneal (i.p.) injection increased the leukocyte count, with statistically significant increases in neutrophils in the peritoneal cavities of rats [152]. It is perhaps not a coincidence that infants < 6 months with extremely low levels of melatonin (27.3 ± 5.4 pg/mL) [139] also exhibited a significant under-activation of IFN and related genes compared to those aged 6–24 months during respiratory viral infections [153]. The unusually high amount of melatonin in children between one and three year of age (329.5 ± 42.0 pg/mL) becomes exceptionally meaningful when considering similar supporting results from in vitro and in vivo work on melatonin pretreatment against viral infections.

The priming and preactivation of the IFN immune response system by melatonin pretreatment and the treatment dose used are directly correlated with survival rates during viral infections. Balb/c mice infected intranasally with the influenza A/PR/8/34 (PR8) H1N1 virus had a higher survival rate (~75%) when pretreated at 6 h with 200 mg/kg melatonin (subcutaneous injection) compared to mice similarly treated but at 48 h post-infection (~40%). However, only 20% of the mice pretreated with 20 mg/kg melatonin or injected with control solvent (both pretreatment and post-treatment) survived [141]. Adult male NMRI outbred mice infected with the Venezuelan equine encephalitis (VEE) virus that were pretreated with melatonin at 500 µg/kg body weight (bw) either starting three days before or at the moment of viral infection achieved 25% survival rate at day 10 post-infection, whereas animals treated with the same level of melatonin 24 h after infection all died by day 7. In addition, doubling the melatonin dose from 500 to 1000 µg/kg bw reduced the mortality rate of VEEV-infected mice from 100% to 16% [154]. Correspondingly, in a multicenter, observational study involving 58,562 hospitalized adult individuals infected by SARS-CoV-2, daily melatonin dose of 2.61 mg was not associated with reduced mortality [155]. Conversely, results from a single-center, prospective, randomized clinical trial reported higher doses of 10 mg melatonin were shown to reduce COVID-19 mortality rates from 17.1% in the control group (standard therapy, n = 76) to 1.2% in the melatonin group (standard therapy + melatonin, n = 82) [156]. Conversely, in a retrospective descriptive case series of patients, 100% of COVID-19 patients (n = 10) given high-dose melatonin (36–72 mg/day per os) in four divided doses as adjuvant therapy all recovered with reduced hospital stay without the need for mechanical ventilation intervention [157]. Therefore, the presence of adequate melatonin before or at the time of infection critically influences the timely suppression of viral infection and the subsequent expression of various viral proteins that can modulate host gene expression to cause IFN evasion and resistance that may result in severe disease progression.

A multi-omic global analysis of infected cells revealed that SARS-CoV-2 expresses viral proteins that extensively remodeled one-third of the RNA-bound proteome (RBPome), involving both upregulation and downregulation of more than 300 RNA-binding proteins (RBPs). The host–virus interaction between cellular and viral RBPs exerted profound effects on RNA metabolic pathways, noncanonical RBPs, as well as antiviral factors. Of the six viral RBPs—ORF1ab, ORF9b, M, N, and S proteins—that interact with viral and cellular RNAs, only open reading frame 1ab (ORF1ab) and the nucleocapsid (N) protein are capable of establishing the most optimal and stable interactions during UV crosslinking with RNAs [158]. ORF1ab, the largest gene in SARS-CoV-2, contains open reading frames that encode polyproteins that are cleaved to yield 16 nonstructural proteins (NSPs) responsible for assembly, transcription, replication, and control of host gene expression [56,159,160]. During viral replication, expression of the N protein alone was sufficient to block IFN induction, while expression of the nonstructural protein 1 (Nsp1) was necessary for the inhibition of IFN signaling [138]. The SARS-CoV-2 Nsp1 is a major pathogenicity factor that interacts with SG-associated proteins, altering host gene expression and protein synthesis to enhance viral replication and suppression of the innate immune system [161,162,163,164]. It is not a coincidence that both N and Nsp1 proteins contain intrinsically disordered regions (IDRs) that facilitate LLPS resulting in the formation of viral molecular condensates critical for replication and infectivity, and that the timely presence of melatonin can dynamically regulate viral molecular condensates formed via LLPS to suppress viral infection and replication.

### 3.3. SARS-CoV-2 Molecular Condensates Are Viral Replication Factories That Enhance Immune Suppression and Evasion

SARS-CoV-2 is a single-stranded positive-sense RNA virus that produces negative-sense RNA when it is replicating in the cytoplasm of infected cells [165]. The first step in the replication of coronaviruses (CoVs) including SARS-CoV-2 is the synthesis of the negative-strand counterpart [166,167]. The N protein is the most copiously expressed protein during viral infections [168] responsible for releasing nascent negative-strand RNA that promotes a template switch that enables the transcription of subgenomic RNAs [169]. The enrichment of IDRs in N protein and association with RNA can promote LLPS in infected cells [170,171,172], causing the N protein to form biomolecular condensates with both homo-polymeric and viral genomic RNA under physiological salt conditions in vitro [173,174]. The protein-RNA electrostatic interactions that stimulate N protein phase separation can be tuned by pH, salt, and RNA concentrations, and enhanced by the prion-like disordered sequences in the N- and C-terminal, as well as the linker IDRs [175]. These dynamic “viral factories” [176,177] formed via N protein LLPS assist in the packaging of the viral genome into distinct ribonucleoprotein (RNPs) complexes [178], which serve as scaffolds to accelerate viral replication through association with other host biomolecular condensates assembled from RBPs including stress granules (SGs), fused in sarcoma (FUS), and TAR DNA-binding protein 43 (TDP-43) [132,175,179,180,181]. LLPS-mediated viral molecular condensates may be the fundamental physicochemical process employed by viruses to increase efficacy of viral replication [182]. The formation of liquid-like, cytoplasmic, membraneless organelles known as viral inclusion bodies (IBs) or “viral factories” where viruses concentrate and replicate in infected cells is extensively reviewed for many single-stranded, negative-sense RNA viruses [174,183]. Viruses that utilize inclusion bodies for de novo RNA synthesis and replication include the Ebola virus (EBOV, EBV) [184], human metapneumovirus (HMPV) [185], influenza A virus (IAV) [186], measles virus (MeV) [187], rabies virus [188,189], respiratory syncytial virus (RSV) [190,191,192], and the vesicular stomatitis virus (VSV) [193,194].

In human airway epithelial cell cultures, after successful attachment and fusion to cytoplasmic membranes, the SARS-CoV-2 N protein induced the formation of IBs in the cytoplasm that were prone to aggregate close to the apical surface. These membraneless viral particles were often found to be enclosed in mitochondria in the cytoplasm [195]. Similar to SARS-CoV-2, the expression of EBV N protein generates dynamic, cytoplasmic IBs responsible for key RNA replication processes during the virus life cycle by recruiting and interacting with important host proteins such as the nuclear RNA export factor (NXF1) [196,197]. Membraneless IBs formed as a result of LLPS can shield newly synthesized viral RNA from innate immune responses and may even sequester specific host proteins, such as stress granules marker proteins in order to disrupt the canonical formation of SGs [198]. The ubiquitous presence of highly disordered regions in viral proteins can also allow many viruses to freely interact with host biomolecular condensates for efficient immune evasion, replication, and persistence.

### 3.4. Interactions between Viral Intrinsically Disordered Regions and Host Biomolecular Condensates Enhance Viral Replication by Exploiting Stress Responses

Intrinsically disordered regions (IDRs) in proteins often lack a well-defined three-dimensional structure [199]. IDRs lack the large hydrophobic amino acids that form structured domains, and can, therefore, conduct rapid exchanges between multiple conformations to assemble condensates without altering the affinity of binding interactions during LLPS [200,201]. Increased cell complexity in eukaryotes is correlated with a significantly higher level of disorder compared to prokaryotes [202], implying the lack of an ordered, three-dimensional structure confers higher flexibility in protein–protein interactions that are instrumental in cell signaling and molecular communication [203,204,205]. Viruses employ several successful tactics to hijack and control host biomolecular condensates by utilizing the unique features of IDRs in their proteins to accomplish this task [206]. Consequently, IDRs in viruses are often associated with viral infectivity and pathogenicity [207]. Many single-stranded RNA viruses, such as *Flaviviridae* and *Picornaviridae*, can localize to host membraneless organelles (MLOs) including the nucleolus [208,209,210], the stress granule [98,99,211,212], and the processing body (P-body) [213,214]. IDR proteins of the *Flaviviridae* family including the ZIKV are involved with shell particle formation, replication, and virulence [215]. Localization to the nucleolus and subsequent disruption of cell division is a common feature of coronavirus N proteins which contain a high level of IDRs [2,216].

The SARS-CoV-2 proteome possesses high structural stability with the exceptions of the N protein and two nonstructural proteins (ORF6 and ORF9b) that are highly disordered [217]. Most SARS-CoV-2 proteins are ordered but can contain disordered regions, such as the Nsp1 C-terminal region (Nsp1-CTR; amino acids 131–180) [218]. Disordered regions in viral proteins can easily bind to host proteins to facilitate replication, while at the same time, modulate host gene expression for antibody escape and immune evasion resulting in increased pathogenicity [217]. Most antigenic sites where variants capable of immune evasion emerge are enriched in IDRs [2,219] and can compromise effectiveness of neutralizing antibodies generated by vaccines [220]. Approximately 51% of experimentally determined IDRs in SARS-CoV-2 are located in the N protein [2], and not surprisingly, sera from mice immunized with nucleocapsid-based vaccines may enhance control of SARS-CoV-2 infections [221]. Even though human coronaviruses are not distinguished for possessing abundant IDRs—~7.3% in the NL63 proteome compared to 77.3% in that of the Avian carcinoma virus [202]—and the SARS-CoV-2 proteome exhibits an extremely high level of structural order with only a few functionally relevant proteins displaying IDRs [217], an extensive examination of the dark proteome of this virus revealed that almost the entire SARS-CoV-2 virus contain molecular recognition features that are important sites for intrinsic disorder-based protein–protein interactions [222]. While further clarification on the effects of the dark proteome on interactions with host MLOs is urgently required, much work has been done to elucidate how NSP1 and N protein IDR interactions with host MLOs result in translational shutdown and immune inhibition/evasion [55,80,161,164,223,224,225,226,227,228,229,230]. By the end of 2020, there was already abundant evidence demonstrating the SARS-CoV-2 N protein can phase separate to form molecular condensates that interfere with human host SG formation [132,175].

### 3.5. SARS-CoV-2 Nucleocapsid Enlists Nonstructural Protein 1 to Shut down Host mRNA Translation and Modulate Expression of IFN Genes

The SARS-CoV-2 N protein containing rich IDRs enhance viral replication by phase separating into high-density membraneless condensates acting as “viral factories” that can recruit the RNA-dependent RNA polymerase (RdRp) responsible for enabling high initiation and elongation rates during viral transcriptions [132,231]. In addition, the N protein can partition into the low-complexity domains and the phase-separated forms of host biomolecular condensates, including SGs, FUS, and TDP-43, hijacking these MLOs to accelerate viral replication [175]. At neutral pH, and moderate salt concentration and temperature, the SARS-CoV-2 N protein is extremely disordered, while phase separation can induce significant changes in the secondary structure of the N protein that may facilitate the assembly of RBPs that package the viral genome within viral molecular condensates [171]. Even in the absence of phase separation, the high IDRs in the N protein can significantly accelerate aggregation of amyloid fibrils in vitro, whereas the structurally stable S protein of SARS-CoV-2 had no effect on ⍺-synuclein aggregation in SH-SY5Y cells [232]. Perhaps not coincidentally, the mean levels of N proteins in neuron-derived extracellular vesicles (NDEVs) isolated from plasma of subjects with PASC and neuropsychiatric (NP) manifestations were significantly higher compared to PASC subjects without NP; resolved, acute COVID-19 subjects without PASC; and healthy controls [233]. Similarly, Neuro-PASC patients exhibit higher T cell responses to the nucleocapsid protein compared with control convalescent patients, supporting the theory that a persistent reservoir of the N protein is responsible for the activation of unique immunological signatures biased towards N proteins in Neuro-PASC individuals [234]. Remarkably, skin biopsies obtained from several PASC patients with symptoms of POTS revealed unusual aggregation of cutaneously phosphorylated ⍺-synuclein amyloid fibrils [235].

In order to successfully package viral genomes during replication, the N protein requires support from other nonstructural proteins that can suppress host gene translation to evade innate immune responses that target and inhibit viral genome replication. The C-terminal residues 131–180 of the nonstructural protein 1 (nsp1) are intrinsically disordered in an aqueous environment and are prone to self-aggregation [218]. The potential binding of nsp1 to mRNA may be responsible for mediating mechanisms behind the successful evasion of host translation shutoff by nsp1 [236,237]. Conformational changes of nsp1 due to electrostatic interactions in the IDRs of nsp1 allow highly flexible and indiscriminate access to binding partners such as host mRNA export receptor heterodimer NXF1-NXT1 and the ribosomal 40*S* subunit [164,218,227]. Widely known as a pathogenic virulence factor, nsp1 effectively shuts down host mRNA translation to prevent expression of IFNs and ISGs by binding with the 40*S* and 80*S* ribosomes to form ribosomal complexes in vitro and in vivo [138,164,238]. At the same time, molecular interactions between nsp1 and NXF1-NXT1 block mRNA translocation to the cytoplasm and subsequent translation by impeding binding of NXF1 to mRNA export adaptors and preventing NXF1 docking at the nuclear pore complex (NPC) [164,227]. Both the SARS coronavirus and the SARS-CoV-2 virus are highly adept at suppressing host protein synthesis by accelerating the degradation of cytosolic cellular mRNAs, in essence, hijacking the host translation machinery to impair the translation of innate immune genes to inhibit antiviral responses that include the IFN signaling system—the first line of defense in vertebrates [56,129,239,240].

**Figure 1:**
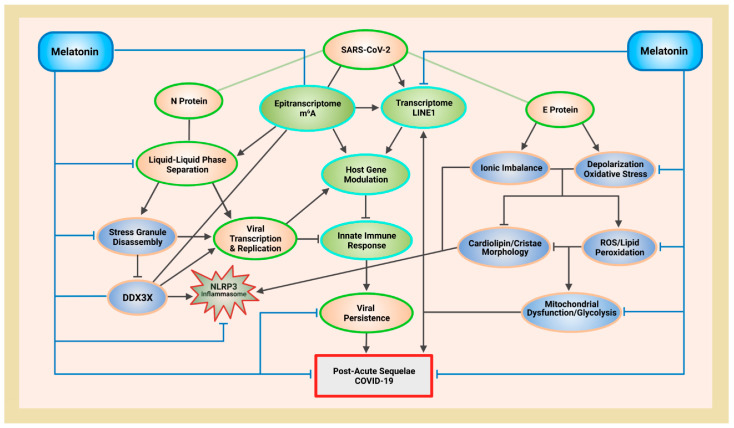
Schematic illustrating melatonin attenuation of acute infection, viral persistence, and post-acute sequelae COVID-19 (PASC) from potential alterations to the epitranscriptome and transcriptome via RNA m^6^A modifications and LINE1 derepression by the SARS-CoV-2 virus. The envelope (E) protein causes extensive mitochondrial distress and elevates oxidative stress via membrane depolarization and ionic imbalances that activate LINE1 derepression, NLRP3 inflammasome apoptotic signaling, stress granule formation, and nucleocapsid (N) protein liquid-liquid phase separation (LLPS). N protein LLPS forms membraneless condensates that not only facilitate viral transcription, genome packaging, and dissemination, but also enhance the suppression of host gene expression to evade innate immune responses via the disassembly of stress granules and the hijacking of DEAD-box RNA helicase DDX3X. Melatonin employs antioxidant-dependent and -independent strategies to modulate m^6^A modifications, suppress LINE1 derepression, rescue mitochondrial dysfunctions, and reduce oxidative stress. Melatonin regulates N protein LLPS to block the sequestration of DDX3X and the formation of NLRP3 inflammasome, as well as the disassembly of stress granules to support innate antiviral immune response, inhibiting viral transcription and replication, maintaining host gene stability and integrity to prevent severe disease and PASC (see Abbreviations for additional acronyms).

Ribonucleic acid (RNA) is a single-stranded molecule with alternating ribose and phosphate groups attached to adenine, uracil, cytosine, or guanine bases [241]. RNA regulates phase separation formation of MLOs by providing multivalency through nonspecific negative charges [242,243]; and the nongenetically coded, reversible, epitranscriptomic modifications in mRNAs play vital roles in stress responses, especially during viral infections [244,245]. Thus, viral *N*^6^-methyladenosine (m^6^A) epitranscriptomic modifications that can change charge, conformation, and anchoring of RNA-binding proteins (RBPs) not only regulate and enhance viral and cellular phase separation [246], but also promote mRNA degradation and/or suppression of mRNA translation, become extremely relevant during viral replication [247,248]. Viral RNA m^6^A can deviously mimic host cellular RNA to assist viruses escape detection by innate immune surveillance [249,250]. Viruses including SARS-CoV-2 effectively exploit m^6^A modifications to suppress interferon signaling and increase viral gene expression. Consequently, a reduction in m^6^A modifications in SARS-CoV-2 or other viruses and host genes enhances downstream innate immune signaling and the expression of IFN genes that drive the type I interferon response [251,252]. Therefore, the ability to modulate viral and host m^6^A modifications and the timely inhibition of N protein phase separation may be critical in the effective dismantling of the viral replication machinery of SARS-CoV-2 and other viruses. Melatonin may be the quintessential linchpin—being an evolutionarily conserved regulator of viral/host LLPS and m^6^A epitranscriptomic modifications—that can reduce viral replication and persistence during viral infection and PASC development (Figure 1).

## 4. Melatonin Is an Ancient Molecule That Can Regulate Virus Phase Separation

Melatonin (N-acetyl-5-methoxytryptamine) is a ubiquitous, mitochondria-targeted molecule present in all tested eukarya and bacteria [253]. In March 2022, the first discovery of the serotonin N-acetyltransferase (*SNAT*) gene—responsible for the penultimate formation of N-acetylserotonin (NAS) [254] before its final conversion into melatonin [255]—in archaea [256] further consolidates the status of melatonin as a regulator of biomolecular condensates in all three domains of life in the cellular empire [257]. Phase separation is an energy efficient thermodynamic process used by living organisms in all three domains of life [57,60,61,62,63] to rapidly respond and adapt to changing environments under stress as a fundamental survival strategy [258,259]. Melatonin is present in many primitive unicellular organisms such as *Rhodospirillum rubrum* (precursor to mitochondria) and the cyanobacteria (precursor to chloroplasts) [260,261]. The fact that cyanobacteria uses adenosine triphosphate (ATP) to regulate the assembly and disassembly of biomolecular condensates in order to conserve energy expenditure during low metabolic activities in the absence of light or ATP production [262] may imply that melatonin exerts distinct modulatory control over phase separation not only in eukaryotes, but also prokaryotes, where condensate formation is tightly correlated with reduced ATP levels from impaired ATP hydrolysis [263].

Melatonin is well recognized for its ability to protect and enhance ATP production in mitochondria of eukaryotic cells [264,265] that acquired melatonin synthetic ability via horizontal gene transfer from prokaryotic cells including the cyanobacteria [261,266,267]. Therefore, early life forms may have utilized melatonin as a potent regulator of host and viral phase separation during stress and viral infection. The reversible assembly of adaptive, evolutionarily conserved, stress-triggered, survival-promoting membraneless condensates are dynamically tuned by ATP, RNA, and/or molecules and processes dependent upon ATP and RNA [64,69,268,269,270]. Thus, it is not unexpected to find LLPS of SARS-CoV-2 N protein to be modulated by both ATP and RNA, and that melatonin may exert unique and significant regulatory controls over viral LLPS.

### 4.1. ATP and RNA Controls N Protein Phase Separation in a Biphasic Manner

The hydrolysis of ATP phosphoanhydride bonds provides free energy to support post-translational modifications including phosphorylation that can either maintain fluid phases or generate supersaturation gradients to initiate phase separation and induce condensate assembly [57,271,272]. However, ATP can also become a biological hydrotrope at physiological ranges between 2 and 8 mM to solubilize LLPS-formed condensates by reducing intermolecular contacts, increasing hydration, and promoting solubility [268,273]. Similarly, the high negative charge densities buried in the phosphate backbones of RNA confer powerful electrostatic forces that can fine-tune the composition and morphological outcome of condensate phases in LLPS [274,275]. LLPS and condensate formation is enhanced by low levels of negatively charged RNA interacting with positively charged proteins, whereas condensates are dissolved by high levels of negatively charged RNA that repel positively charged proteins [270]. Thus, how an organism employs melatonin to control the level of ATP concentration and modify RNA properties to effectively tune the size, shape, viscosity, and composition of biomolecular condensates [268,276] directly affects the health and survival of the organism.

In 2021, Dang et al. showed for the first time that ATP modulates SARS-CoV-2 N protein phase separation in a biphasic manner, and that ATP is capable of completely dissolving viral condensates formed by N protein LLPS at molar ratios of 1:500 (N-protein:ATP). Conversely, droplets began to assemble at a lower molar ratio of 1:25 and continued to increase in number and size with increasing ATP, but only up to a concentration of 1:200, beyond which additional ATP actually reduced droplet numbers until all condensates formed were totally dissolved at 1:500 molar ratio [277]. In the same manner, RNA and the IDR in SARS-CoV-2 N protein drive phase separation in an RNA-dependent manner. N protein and nonspecific 17-mer ssRNA could phase separate into condensates only within a defined range of RNA concentration, where 10 μM N protein induced maximal phase separation together with 5 μM 17-mer RNA, whereas further increases in RNA concentration inhibited phase separation [278]. The addition of a longer 24-mer poly(A) (A24) RNA at a molar ratio of 1:0.5 (N-protein:RNA) also increased N protein phase separated droplet size and turbidity value to ~2 µM and 0.97, respectively. However, to dissolve droplets formed by LLPS of N protein and A24, a much higher level of ATP at a molar ratio of 1:750 (N-protein:ATP) was found to be necessary [277].

### 4.2. Elevated Extracellular ATP May Reduce Viral Replication

Extracellular ATP reduces viral replication in the vesicular stomatitis virus (VSV), Newcastle disease virus, murine leukemia virus, and herpes simplex virus (HSV) [279]. Elevated extracellular ATP is a part of the host danger signal response [280], and elevated ATP release is not uncommon during viral infection in vitro and in vivo. Similar to ATP synthase dimers that localize exclusively to high-curvature cristae invaginations of the inner mitochondrial membranes (IMMs) [281], the production and hydrolysis of extracellular ATP are also dependent upon the structural integrity of high-curvature caveolae and lipid raft domains where ATP synthases and ATPases are commonly localized [282,283]. Severe COVID-19 in children is rare. Compared with healthy controls, children with acute COVID-19 infections, whether severe or mild, all exhibited higher plasma levels of ATP that were negatively correlated with the frequency of regulatory T cells but positively correlated with the frequency of CD4+ T cells [284]. Conversely, the level of CD4+ T cells—regarded as a biomarker of protective immunity [285]—is usually significantly reduced in adults with severe or critical COVID-19 compared to healthy controls [286]. Melatonin protects both mitochondrial and extracellular ATP production by maintaining curvature and ensuring structural integrity of lipid domains where ATP synthases and ATPases are located [287]. The ability of SARS-CoV-2 to disrupt ATP synthases, and its efficacy in modulating mitochondrial dynamics and metabolism to evade host immune response, enhance replication, and establish viral persistence may be attenuated by the well-timed presence and/or application of adequate melatonin [288].

## 5. Melatonin Protects Mitochondria and ATP Production to Inhibit N Protein Phase Separation

Mitochondria are the “energy powerhouse of the cell” that control respiration and ATP synthesis [289,290], and mitochondria are directly targeted by viruses during infection to facilitate the modulation of cellular metabolism and innate immunity [291]. The fundamental features of optimal mitochondrial dynamics are characterized by the ability to connect and elongate (fusion), divide (fission), and turnover (mitophagy). Disruption of mitochondrial bioenergetics during viral infections may explain how RNA viruses hijack mitochondrial dynamics to support viral replication and persistence [292]. Both the hepatitis B and hepatitis C viruses promote chronic liver damage by altering the balance of mitochondrial dynamics towards fission and mitophagy in order to reduce virus-induced apoptosis, thereby enhancing viral persistence [293,294]. The SARS-CoV-2 virus relies on a sophisticated, multipronged approach to commandeer and manipulate mitochondrial dynamics and metabolism, evading mitochondria-dependent immune response to promote viral replication and pathogenesis [295]. The SARS-CoV-2 dsRNA, which is an intermediate of positive-strand RNA virus replication, has been found to localize in mitochondria [296], while computational modeling of SARS-CoV-2 viral RNA subcellular localization revealed much stronger transcript residency signals toward the mitochondrial matrix and nuclear compartments compared to other coronaviruses [297]. An analysis of changes in molecular composition of mitochondria captured by Raman microspectrometry and biomolecular component analysis (BCA) algorithm found a marked reduction in mtDNA content in microglia treated with spike protein or heat-inactivated SARS-CoV-2 virus [298].

Integrative imaging techniques provided evidence of extensive alterations to cellular organelles, including significant fragmentation of the Golgi apparatus and perturbation of mitochondrial morphology and function. Mitochondria in cells infected by SARS-CoV-2 displayed swollen cristae and matrix condensation, together with significant decreases in mitochondrial ATP synthase subunit 5B (ATP5B) that implies metabolic rewiring away from oxidative phosphorylation in favor of glycolysis [299,300]. The SARS-CoV-2 virus enhances replication by causing mitochondrial dysfunction via membrane depolarization and mitochondrial permeability transition pore (mPTP) opening in a time-dependent manner, with more damage observed at 12 h post-infection compared to 3 h. In order to prevent clearance and degradation of damaged mitochondria, the SARS-CoV-2 virus stalls initiated mitophagy to suppress mitochondrial quality control and clearance of virus by inhibiting binding of mitophagy mediator LC3 and its binding adaptor protein p62 [296,301]. In diabetic cardiomyopathy (DCM), the clearance of dysfunctional mitochondria by mitophagy is often impaired. In a DCM mouse model, melatonin supplementation at 20 mg/kg/day for 4 weeks increased the expression of both LC3-II and p62, resulting in upregulated Parkin-mitophagy that increased clearance of dysfunctional mitochondria to restore mitochondrial quality control [302].

### 5.1. Melatonin Rescues Mitochondrial Membrane Potential from SARS-CoV-2 Envelope Protein-Induced Depolarization

RNA viruses and bacterial infections promote ion channel activities, resulting in membrane depolarization that can activate pro-inflammatory, apoptotic NLR pyrin domain containing 3 (NLRP3) inflammasomes that are a major source of inflammatory IL-1β and IL-18 cytokines [303,304,305,306]. The SARS-CoV envelope (E) protein is a viroporin that regulates host cell microenvironment including pH and ion concentrations, causing death in humans and animal models by inducing the pro-inflammatory NLRP3 inflammasome response [307,308,309]. Using similar mechanisms, the SARS-CoV-2 E protein also increases pathogenicity by forming a homopentameric cation channel to modify host ion channel homeostasis in support of viral replication [310,311,312,313]. Mutations of the E protein can enhance the open channel conformation in ion-channel functionality, causing increased virulence and pathogenicity that are correlated with high COVID-19 mortalities [314]. Ion channels formed by viroporins not only allow water and ions to penetrate cell membranes [315], but also generate progressive membrane permeation and damage, disrupting membrane potential and collapsing ionic gradients that facilitate viral budding and release, spreading the virus to surrounding cells [316,317]. Molecular dynamic simulations demonstrated that the E protein can promote viral replication by reducing intracellular calcium in transfected cells and enhance viral budding by bending surrounding lipid bilayers [318].

#### 5.1.1. Membrane Depolarization Impairs Oxidative Phosphorylation and Cation Homeostasis

Mitochondria infected by SARS-CoV-2 display swollen cristae [299,319,320]. Modulations to cristae topology directly affects mitochondrial function and bioenergetics [321]. ATP synthesis during oxidative phosphorylation (OXPHOS) in mitochondria is dependent upon the F_1_F_0_ ATP synthase (complex V) of the electron transport chain (ETC) to drive proton re-entry powered by chemical energy maintained by the negative membrane potential (ΔΨm) of inner mitochondrial membrane (IMM) consisting of inner boundary membranes (IBMs) and cristae—the principal site of oxidative phosphorylation in mitochondria [322,323,324,325]. Changes in the ΔΨm—depolarization or hyperpolarization—by a decrease (less negative) or an increase (more negative) of the ΔΨm, respectively, can alter mitochondrial homeostasis and bioenergetics [322]. Proper ΔΨm of IBM maintains a strong electrical force to keep protons close to cristae membrane within the intercristal space (ICS; cristae lumen) [326,327,328]. Depolarization of the mitochondria membrane can cause a partial or complete collapse of the ΔΨm [329], resulting in dysfunctional, swollen, unfolded cristae that no longer can maintain optimal ATP production via OXPHOS [330]. Decline of the ΔΨm causes matrix condensation, leading to the unfolding of cristae which expands matrix volume to cause mitochondrial swelling [331,332]. Decreased ΔΨm reduces ATP production by lowering ETC activities, but targets damaged areas for clearance by mitophagy [333,334,335]. Yet, inhibition of mitophagy by SARS-CoV-2 prevents the timely clearance of dysfunctional mitochondria that prevents higher ATP production via OXPHOS in favor of glycolysis [300,336,337,338].

Membrane depolarization from viroporin ion channel activities can elevate production of reactive oxygen species (ROS) via increased matrix pH due to cation influx and/or anion efflux [339]. Depolarization opens different types of voltage-gated calcium channels (VGCCs) in a wide range of cell types including both excitable and nonexcitable cells [340,341]. Opening of VGCCs allows the rapid influx of extracellular calcium (Ca^2+^) that serves as electrical signaling messengers to initiate different important cellular processes [342]. Viruses—including the poliovirus [343], alphavirus [344], human immunodeficiency virus type I (HIV-1) [345], influenza virus [346], SARS-CoV [347], and SARS-CoV-2 [310]—encode viroporins to form ion channels in host cell membranes that facilitate membrane permeability to promote viral entry, replication, release, and dissemination to surrounding cells [347]. Dysregulated calcium signaling may underlie autonomic dysfunctions [348,349] often associated with PASC [350,351,352], including postural orthostatic tachycardia syndrome (POTS) [353,354]. Unlike viroporins of other viruses that increase intracellular Ca^2+^ by modulating plasma membrane permeability [355,356], the SARS-CoV-2 E protein can decrease Ca^2+^ content in transfected cells by ~61.5% (0.1286 ± 0.0745 AU, N = 22) compared to nontransfected cells (0.2002 ± 0.096, N = 19; *p* = 0.01), indicating potential leakage, suppression, or sequestration of Ca^2+^ by the virus. Secondary osteoporosis often occurs with PASC, where a decrease in bone mineral density (BMD) by a mean of 8.6% (± 10.5%) could be detected in COVID-19 at a mean of 81 (± 48) days after hospital discharge. This significant loss in BMD far exceeded normal age-related annual BMD loss, resulting in a two-fold increase in the osteoporosis ratio [357].

Furthermore, the SARS-CoV-2 E protein is localized intracellularly and may be responsible for proton efflux in transfected cells [318]. An acidic pH can adjust the conductivity and ion selectivity of the ion-conducting transmembrane domain of E protein by protonating the Glu8 side chain carboxyl, altering the carboxy-terminal conformation [312]. The influenza B virus viroporin proton channel is pH-gated and mediates virus uncoating when activated by acidic pH [358]. Ionic imbalances in cells affecting the homeostasis of cations, including calcium (Ca^2+^), magnesium (Mg^2+^), zinc (Zn^2+^), potassium (K^+^), and sodium (Na^+^), can interfere with innate and adaptive immunity that affect the pathogenicity of viruses [307,359,360,361].

#### 5.1.2. Viroporin Ion Channel Activities May Regulate Virus Phase Separation

Potassium (K^+^) efflux triggers the activation of the NLRP3 inflammasome upon infection by RNA viruses [303], including SARS-CoV-2 [362,363], where elevated urinary loss of potassium is often associated with COVID-19 disease severity [364]. Experimental work showed the SARS-CoV-2 ORF3a viroporin priming and activation of the NLRP3 inflammasome were dependent upon K^+^ efflux [365]. K^+^ is a rate-liming modulator of the glutamate transport cycle, where intracellular K^+^ relocates the glutamate binding site to the extracellular side of the membrane, and extracellular K^+^ induces glutamate release upon transporter relocation [366]. Glutamate promotes LLPS of the *Escherichia coli* single-stranded-DNA binding protein [367]. Thus, K^+^ efflux that can elevate glutamate availability [368] may enhance SARS-CoV-2 phase separation. Indeed, altered glutamine metabolism and dependence on glutamine receptor subtype 2 for internalization are associated with SARS-CoV-2 infections [369,370]. Mitochondrial dynamics dysfunction and Ca^2+^ dysregulation as a result of membrane depolarization induced by viroporin ion channel activities can also affect leucocyte functionality to suppress and evade immune responses during SARS-CoV-2 infection to enhance viral phase separation for viral replication.

### 5.2. Melatonin Attenuates Membrane Depolarization and Balances Ion Homeostasis by Antioxidant-Dependent and -Independent Mechanisms to Protect Mitochondria and Lymphocytes during Viral Infection and PASC

Leukocytes of patients recovered from COVID-19 presented loss of mitochondria membrane potential (ΔΨm) even at 11 months post-infection [371]. Leukocytes are responsible for the production of first line IFN-⍺ immune response [150,151], and the loss of ΔΨm caused by viroporin-mediated membrane depolarization may be one of the most important underlying causes for the development of PASC [371]. Lymphopenia and the depletion of T lymphocyte subsets were found in 98% (153/157) of patients infected by SARS-CoV in 2003 without any preexisting hematological disorders [372]. Correspondingly, patients infected by SARS-CoV-2 are associated with persistent lymphopenia [373,374] and functional exhaustion of lymphocytes [375]. COVID-19 disease progression is correlated with a nearly three-fold increased risk of severe COVID-19 (random effects model, OR = 2.99, 95% CI: 1.31–6.82) [376], while low lymphocyte counts in patients are deemed to be effective predictors of disease severity and hospitalization [377,378].

T lymphocytes are dependent upon functional mitochondria to supply local ATP and to maintain Ca^2+^ homeostasis and signaling during all stages of immune response [379,380]. In T lymphocytes, expression of 75% of the genes associated with survival and proliferation are dependent upon Ca^2+^ influx [381], while mitochondrial dynamics often affect T lymphocyte chemotaxis, where mitochondrial fusion protein OPA1 inhibits lymphocyte migration and chemotaxis, but fission enhances both migration and chemotaxis [382]. It is perhaps not a coincidence that depolarization of mitochondrial membranes can activate dynamin-related GTPase OPA1-dependent fusion to inhibit lymphocyte chemotaxis [383], and that the E protein viroporin can deplete intracellular Ca^2+^ content [318]. Stimulation of T lymphocytes triggers immediate accumulation of active mitochondria with elevated Ca^2+^ influx and heightened OXPHOS, which can also cause transient collapse of ΔΨm due to intense ETC activities, ion flux, and ATP release across the mitochondrial membrane [379]. Thus, inability to repolarize ΔΨm results in a reduction of ATP generation from the loss of electrochemical potential that maintains the gradient that drives the F_1_F_0_ ATP synthase (complex V) [333,384]. Moreover, membrane depolarization also prevents store-operated Ca^2+^ influx after store depletion [385]. Cell sorting experiments revealed that mtDNA damage occurs only in human fibroblast cells with low ΔΨm sustained for 24 h. These cells exhibited continuous, elevated production of hydrogen peroxide (H_2_O_2_) that potentially accentuated a feed-forward cascade of increasing ROS that impaired repair responses and increased mtDNA lesions, resulting in apoptosis [386]. Taken together, membrane depolarization by E protein suppresses not only ATP-dependent purinergic signaling that supports T lymphocyte immune response functions, but also T lymphocyte-mediated expression of genes that are dependent upon Ca^2+^ influx [379,381]. In its multipronged strategies against the SARS-CoV-2 virus, melatonin not only promotes the production of leukocytes [152], but also attenuates membrane depolarization to protect lymphocyte functionality (Figure 1).

Melatonin is a pleiotropic molecule that can maintain optimal membrane potential by either increasing or reducing ΔΨm for maximum efficiency. In hyperpolarized, prorenin-treated microglia, treatment with 100 μM melatonin reduced ΔΨm and attenuated hyperpolarization and ROS overproduction [387]. Conversely, in mitochondria of human oocytes, 10 µM melatonin treatment decreased excessive intracellular Ca^2+^ levels to restore mitochondrial function and significantly increased membrane potential compared to control levels [388], while 1 μM melatonin added to post-thawed equine sperm increased mitochondrial membrane potential and improved mitochondrial function [389]. Membrane depolarization prevents store-operated Ca^2+^ influx after store depletion [385], disrupting T lymphocyte-mediated gene expressions [381]. However, treatment with 500 µM melatonin markedly elevated cytosolic calcium in human platelets by evoking store-operated calcium release from platelet mitochondria [390]. An analysis of human neutrophil respiratory burst and membrane potential changes found melatonin to increase depolarization at concentrations up to 0.5 mM, whereas 2 mM melatonin concentration decreased ΔΨm in neutrophils activated by phorbol 12-myristate 13-acetate (PMA) [391]. Mitochondrial inner membrane depolarization in human HaCaT keratinocytes irradiated with UVB radiation (50 mJ/cm^2^) was normalized by preincubation with 0.01 mM to 1 mM melatonin via the reduction of mitochondrial ROS (mROS) and inhibition of mitochondrial permeability transition pore (mPTP) opening [392].

Viroporin-induced membrane depolarization elevates production of ROS via ionic imbalances from dysregulated cation influx and/or anion efflux [339]. The SARS-CoV-2 virus can also escalate ROS release in Vero E6 cells via opening the mPTP, causing subsequent depolarization and further oxidative stress damage in a time-dependent manner [296,393]. In a self-perpetuating positive feedback loop, oxidative stress from unneutralized excess ROS leads to even more rapid depolarization of the inner mitochondrial membrane potential and subsequent disruption of OXPHOS and ATP production. Damaged mitochondria continue to produce more ROS, resulting in the dreaded ROS-induced ROS release (RIRR) loop [394]. ROS can also cause physiological lipid peroxidation [395], where oxidants attack the carbon-carbon double bond in lipids, initiating a cascading chain reaction that terminates in the formation of reactive aldehyde end products including 4-hydroxynonenal (HNE) [396]. In a pilot study of 21 critically ill COVID-19 patients admitted to the ICU, the only difference in clinical or laboratory parameters monitored between the 14 patients who recovered and the 7 who passed away was the significantly higher level of HNE-protein adducts (*p* < 0.05) obtained from the plasma of the deceased patients compared to levels in survivors during the initial 1–3 days in hospital [397].

Melatonin and its metabolites are potent inhibitors of lipid peroxidation cascades and are extremely effective at scavenging different types of ROS [287,398,399,400,401,402,403]. In leukocytes irradiated with 750 mJ/cm^2^ UVB light (280–360 nm, max: 310 nm), treatment with melatonin suppressed ROS directly in a dose-dependent manner where 10 mM melatonin reduced ROS formation in leukocytes by 260-fold, while 7.5 mM and 5 mM reduced ROS by 120- and 60-fold, respectively [404]. In addition to decreasing ROS via antioxidant-dependent mechanisms [405,406], the regulation of depolarization by melatonin may be via an ionic-based, antioxidant-independent mechanism. The repolarization of gonadotrophin-releasing hormone (GnRH)-induced membrane depolarization in neonatal rat pituitary cells by melatonin could be mediated through the inhibition of Ca^2+^ influx or a hyperpolarization mechanism that is sodium-dependent, involving modulation of the Na^+^/K^+^-dependent ATPase [407]. Jurkat cells undergo apoptosis from anti-Fas-induced mitochondrial membrane depolarization where inhibition of the Na^+^/K^+^ ATPase prevented membrane repolarization via the suppression of monovalent ion movements, particularly the intracellular accumulation of Na^+^ during sustained depolarization without repolarization [408].

Melatonin is an osmoregulator with pleiotropic effects on plasma sodium concentration in animal models [409,410]. This ancient molecule is indispensable in maintaining ion homeostasis in plants [411,412,413], and its comprehensive role as a “broad-based metabolic buffer” includes rhythmic circadian modulation of the Na^+^/K^+^-ATPase as well as the Na^+^/H^+^ exchanger ion-transport activities in human erythrocytes via antioxidant-dependent and -independent mechanisms [414,415]. Both Na^+^/K^+^-ATPase and Na^+^/H^+^ can influence transmembrane chemical gradients [416,417], as well as cytosolic pH and ionic balance [418,419,420]. Therefore, it is not inconceivable that melatonin can adjust salt homeostasis via Na^+^/K^+^-ATPase to regulate LLPS during viral infections as high salt or extremely low salt concentration can inhibit LLPS [421]. Hyponatremia where plasma sodium concentration is below 135 mmol/L is often associated with viral infections including COVID-19 [422]. Furthermore, in vitro experiments found 1.5% NaCl solution can achieve 100% inhibition of SARS-CoV-2 replication in nonhuman primate kidney Vero cells, while 1.1% of NaCl can inhibit viral replication by 88% in human epithelia lung Calu-3 cells [423].

The Na^+^/K^+^-ATPase is a P-type ATPase that utilizes energy from ATP hydrolysis to pump ions across membranes generating an electrochemical gradient [424]. Nonmitochondrial ATPases including P-type Na^+^/K^+^-ATPases are often localized in lipid raft microdomains in lipid bilayers of plasma membranes [425,426,427]. Increased ROS from oxidative stress can reduce membrane fluidity and performance of Na^+^/K^+^-ATPases [428,429,430,431]. Melatonin maintains membrane fluidity by inhibiting lipid peroxidation cascades in an antioxidant-dependent manner [398,399,432,433,434], while its ability to stabilize liquid-ordered (L_o_)-liquid-disordered (L_d_) phase separation in lipid bilayers (tested over a range of temperatures up to 45 °C) preserves necessary lipid raft composition and nanoscopic structure to support various ATPase activities, including those of Na^+^/K^+^-ATPases [414,435].

An analysis of information obtained from various neutron scattering techniques accessing membrane structure and dynamics from SARS-CoV-2 protein–host interactions revealed that molecular interactions during spike protein fusion peptide binding events could induce changes in membrane fluidity and rigidity where fusion peptide 1 increased rigidity while fusion peptide 2 reduced fluidity [436]. Other morphological changes induced by SARS-CoV-2 as a result of fusion events include modification of both lipid composition and membrane structure to produce non-lamellar cubic membranes that facilitate membrane fusion during viral infection [437]. The oxidation of high curvature lipids such as cardiolipin (CL) can result in the rearrangement of lipids in plasma membranes from a fluid lamellar phase to a non-lamellar cubic phase that can impact membrane integrity and stability. The fact that cubic membranes are usually found in membranes with high intrinsic curvature, such as mitochondrial inner membranes with deep cristae invaginations formed by high-curvature lipids that host ATP synthase dimers [281,438,439], further explains how SARS-CoV-2 and other viruses modulate mitochondrial function to favor glycolysis over OXPHOS.

### 5.3. Melatonin Protects Mitochondria Cristae Morphology and ATP Production via Antioxidant-Dependent and -Independent Mechanisms

Phase separation of SARS-CoV-2 N protein may be biphasically modulated by ATP where ATP can completely dissolve viral condensates, which promote pathogenicity and replication, formed by N protein LLPS at molar ratios of 1:500 (N-protein:ATP), but enhance assembly of condensates from low molar ratios of 1:25 up to 1:200 [277]. Hence, mechanisms associated with viral fusion and enhanced viral replication involve targeting of mitochondrial bioenergetics and the production of ATP. An analysis of bulk RNA-seq datasets from COVID-19 patients and healthy controls revealed a marked reduction of mtDNA gene expression in various types of cells including the immune system, with concomitant elevation of genes expressing glycolytic enzymes, and ROS production [336], while an interactome analysis identified multiple mitochondrial proteins that interact with the SARS-CoV-2 N protein [440]. Elevated glucose and sustained aerobic glycolysis in monocytes of COVID-19 patients are directly responsible for boosting viral replication, causing increased NLRP3 inflammasome and cytokine production, inhibition of T proliferation, and apoptosis of lung epithelial cells [338,441]. Metabolic alterations in live peripheral blood mononuclear cells (PBMC) obtained from patients with COVID-19 showed extensive mitochondrial dysfunction with compromised respiration but increased utilization of glucose serving as primary substrate for energy production in place of OXPHOS [442]. Substituting OXPHOS ATP production with aerobic glycolysis may lead to a more than 16-fold reduction of ATP. The theoretical maximum of ATP calculated from simultaneous measurements of oxygen consumption and extracellular acidification showed OXPHOS to yield 31.45 ATP/glucose (maximum total yield 33.45), whereas glucose yields only 2 ATP/glucose [443]. Considering ATP can completely dissolve N protein phase separation condensates at concentrations 2.5- to 20-fold above assembly concentrations, with disassembly starting beyond 8-fold increases, it is not surprising that the timely application of melatonin can effectively suppress viral replications.

#### 5.3.1. Melatonin Suppresses Aerobic Glycolysis to Enhance Oxidative Phosphorylation

Melatonin is a powerful glycolytic that can inhibit aerobic glycolysis (the “Warburg effect”) by steering pyruvate metabolism towards the citric acid (tricarboxylic acid, Krebs) cycle and OXPHOS, and avoiding aerobic fermentation of glucose by glycolysis [444,445,446]. Melatonin can enhance mitochondrial OXPHOS ATP production [265] by different mechanisms including the stimulation of the SIRT3/PDH axis to reverse the Warburg phenotype in lung cancer cells in vitro [447]; and the suppression of hexokinase-2 overexpression to ameliorate glycolytic overload, improving mitochondrial ATP production and normalizing glycolysis to protect mitochondrial function in chronic kidney disease mesenchymal stem/stromal cells [448]. The SARS-CoV E protein ion channel induces membrane permeabilization that decreases ΔΨm in mitochondrial inner membranes [315,371]. Loss of membrane potential not only reduces ATP production due to impaired OXPHOS, but can induce the production of even more ROS due to accumulation of reducing equivalents from lower ETC activities that result in the creation of reductive stress that continues generate additional ROS to perpetuate the RIRR positive feedback loop [322,394,449,450]. The generation of excess ROS during SARS-CoV-2 infection [296] can initiate powerful lipid peroxidation cascades that damage lipid composition of the cristae, resulting in loss of ATP synthase function.

#### 5.3.2. Melatonin and Metabolites Preserve Cardiolipin Function in Cristae by Preventing Lipid Peroxidation Cascades

The apex of deep IMM cristae invaginations provides the ideal location for hosting dimerized ATP synthases of eukaryotic mitochondria [281,451]. Dimerized ATP synthases are seven-fold more active than ATP monomers [452], and dimerization of ATP synthases is a prerequisite for shaping the high curvature cristae structure [453,454]. The deep negative membrane curvatures at the apexes of cristae are maintained by the unique cone-shaped structure of cardiolipin (CL) that not only increases bending elasticity of the IMM but also the regulation of formation and stability of respiratory chain complexes [455,456,457,458]. Accordingly, mitochondrial membranes can comprise up to 25% CL [459,460]. CL is a negatively charged, dianonic lipid that can dramatically lower pH at membrane interfaces to increase proton (H^+^) concentration (~700 to ~800) [461,462] to elevate ATP production [463]. The oxidation of just one fatty acid chain in CL can lead to vast conformational changes in the entire molecule, resulting in reduced membrane thickness, and potential impairment of proton and electron transport that are dependent on CL-mitochondrial protein interactions [464,465]. Elevation of ROS as a result of depolarized mitochondrial membranes during viral infection may increase peroxidation of cardiolipin. The destabilization of mitochondrial supercomplexes as a result of CL peroxidation affects mitochondrial bioenergetics, leading to impaired OXPHOS, reduced ATP production, and other mitochondrial dysfunctions in different tissues manifested in a range of pathophysiological conditions including heart ischemia/reperfusion, heart failure, diabetes, and Barth syndrome [466,467,468,469,470,471,472]. In *Saccharomyces cerevisiae*, disruption of the CRD1 gene responsible for encoding CL synthase resulting in the absence of CL in mitochondria membranes led to a loss of mitochondrial ΔΨm and mitochondrial genome when cultured at prolonged elevated temperature of 37 °C [473]. Interestingly, circulating anticardiolipin antibodies (aCL), which may cause endothelial dysfunction and elevated IgA-aCL, is often associated with increased ischemic burden in patients with coronary artery disease (CAD) [474].

Critically ill COVID-19 patients with coagulopathy and thrombocytopenia often manifest the presence of anticardiolipin antibodies in serum [475]. A meta-analysis and systematic review of 21 studies with 1159 hospitalized COVID-19 patients discovered the presence of antiphospholipid antibodies in ~50% of the patients. Severe disease was correlated with a higher prevalence of aCL (IgM or IgG) compared to noncritical disease (28.8% vs. 7.10%, *p* < 0.0001) [476]. Oxidized LDL bound by anti-lipoprotein antibodies are correlated with IgG-aCL and IgM-aCL [477]; thus, the presence of elevated aCL and other antiphospholipid antibodies is indicative of systemic lipid peroxidation, which may then explain the development of thromboses in the absence of correlated D dimer levels in about one-third of severely ill COVID-19 patients [474,478]. In fact, elevated lipid peroxidation is the only oxidative stress biomarker that is significantly different between intubated COVID-19 patients and/or those who died compared to patients with mild disease. In addition, patients whose lipid peroxidation rose above 1948.17 μM were either intubated or died 8.4 days earlier on average (mean survival time 15.4 vs. 23.8 days) [479]. Melatonin is a potent antioxidant that can protect mitochondrial function by neutralizing ROS to inhibit CL peroxidation [480]. The addition of 10 μM melatonin to rat heart mitochondria almost entirely prevented membrane depolarization induced by Ca^2+^/tert-Butylhydroperoxide (t-BuOOH), a peroxidation promoting peroxide, in addition to reversing cytochrome c release, and mitochondrial matrix swelling [400]. The reason why melatonin is uniquely suited to prevent lipid peroxidation cascades is in large part due to its preferential localization at hydrophilic/hydrophobic membrane interfaces.

Melatonin is uncharged in the entire pH range [481]. Even though melatonin is nonpolar, it can form strong H-bonds with hydrophilic lipid headgroups at hydrophilic/hydrophobic membrane interfaces [482]. Thus, melatonin becomes an efficient scavenger of both aqueous and lipophilic free radicals as a result of the presence of both hydrophilic and lipophilic moieties in the melatonin molecule [483]. As such, melatonin and its metabolites easily neutralize both the hydroxyl radical (^•^OH) and the hydroperoxyl radical (^•^OOH) [484,485]—two dominant ROS molecules that can initiate and sustain chain oxidation reactions of unsaturated phospholipids including CL in plasma membranes [486,487] and mitochondria [488,489]. During viral infections, ionic imbalances from viroporin ion channel activities activate the pro-inflammatory NLRP3 inflammasome which mediates the production of cytokines that can contribute to severe pathophysiology and disease [305,490]. Heightened expression of the NLRP3 inflammasome was detected in leukocytes in the lungs of all patients who did not survive COVID-19 [491]. Melatonin targets NLRP3 inflammasome-mediated cytokine release employing antioxidant-dependent and -independent mechanisms [492].

### 5.4. Melatonin Targets NLRP3 Inflammasomes via Cardiolipin and DDX3X

Cellular stress and dysfunction triggers prionoid-like phase transition of the NLR pyrin domain containing 3 (NLRP3) inflammasome to assemble supramolecular complexes responsible for mediating immune responses, including the release of inflammatory cytokines—IL-1β and IL-18 [493,494,495,496,497]. The NLRP3 inflammasome is a multiprotein complex comprising the NLRP3 sensor, the apoptosis-associated speck-like protein containing a C-terminal caspase recruitment domain (ASC) adaptor, and the caspase-1 (CASP1) protease [498,499]. The activation of NLRP3 inflammasomes is inextricably linked to various types of cell death, including pyroptosis, apoptosis, necroptosis, and ferroptosis [498]. Elevated ROS and mitochondrial distress translocate CL from the inner mitochondrial membrane (IMM) to the outer mitochondrial membrane (OMM) [500], and NLRP3 must be primed and directly bound by externalized CL before it can be activated [501]. As discussed in Section 5.1, viroporin ion channel activities activate NLRP3 inflammasome, and COVID-19 severe pathology resulting from an overactive immune-inflammatory response can be exacerbated by the activation of NLRP3 in infected macrophages in humanized mouse model of COVID-19 [502]. The SARS-CoV-2 E protein viroporin increases NLRP3 inflammasome activation in both murine and human macrophages in a biphasic manner [503] by first suppressing NLRP3 inflammasome activation to aid viral replication leading to advanced disease states that promote the activation of NLRP3 inflammasomes [503]. The activation of NLRP3 inflammasome is often associated with the development of severe COVID-19 [504,505,506] and increased oxidative stress [507], while the production of inflammatory cytokines, including IL-β, may fuel the development of cytokine storms and excess oxidative stress to complete a positive feedback cycle [508,509,510,511,512] that enhances N protein LLPS [513]. This unique biphasic effect may be a reflection of how the SARS-CoV-2 virus interacts with DDX3X and SGs during viral replication (Figure 1).

The regulation of the prionoid transition of NLRP3 inflammasome into supramolecular complexes is mediated by DEAD-box helicase 3 (DDX3X or DDX3)—a host X-chromosome encoded DEAD-box RNA helicase that is often hijacked by SARS-CoV-2 and other viruses [514,515]. In total, 18 species of virus from 12 genera—including the dengue virus [516], HIV-1 virus [517,518], hepatitis C virus [519], Japanese Encephalitis virus, and the Zika virus [520]—have been determined to be dependent upon DDX3X for virulence [521]. The ATP-bound form of DDX3X is necessary for the scaffolding of the ASC domain to transition into irreversible, stable, and insoluble supramolecular prionoid-like assemblies [494]. DDX3X is also a critical regulator of SGs requisite for proper SG maturation [522]. Therefore, the formation of SGs and the assembly of NLRP3 inflammasomes become mutually exclusive, since both SGs and NLRP3 inflammasomes compete for the same DDX3X. Consequently, loss of DDX3X will inhibit both SGs maturation and the scaffolding of ASCs to disrupt NLRP3 inflammasome supramolecular assembly [523,524], while the disassembly of SGs may encourage the aggregation of NLRP3 inflammasomes. Lipid peroxidation that can translocate CL from the IMM to OMM is regarded as a hallmark of severe COVID-19 [479]. Monocytes from severe COVID-19 patients exhibit elevated, persistent presence of ROS and lipid peroxidation compared to mild disease and health controls. The level of lipid peroxidation is strongly correlated with CASP1 activity and ASC aggregate formation, responsible for the NLRP3 inflammasome-dependent IL-β secretion by monocytes [525].

Melatonin targets DDX3X to regulate and enhance innate antiviral responses that suppress viral replication. Viral infection induces cellular stress and mitochondrial distress that activates the host ISR resulting in the formation of SGs. The timely, adequate presence of melatonin can reduce ROS and lipid peroxidation to prevent the translocation of CL to OMM, thus inhibiting the activation of NLRP3 and its prionoid phase transition to form inflammasome supramolecular complexes [492,526,527,528,529]. This effectively allows DDX3X to accelerate the formation and maturation of SGs that can enhance antiviral innate immune signaling [78] and also inhibit viral protein accumulation and replication [66,79]. As such, viruses including SARS-CoV-2 have evolved sophisticated mechanisms to hijack DDX3X by disrupting SG formation. The SARS-CoV-2 N protein not only phase separates to form “viral factory” condensates [176] that protect the viral genomic RNA by packing them into distinct RNP complexes [132,178], but also acts as the central hub for DDX3X interactions [530]. In Vero E6 cells infected by SARS-CoV-2, mass spectrometry analysis revealed DDX3X localizes with viral RNA foci in cytoplasm, and enhances viral infection via interactions with N protein [531]. The fact that the immunopurified complexes were harvested 24 h post-infection may also imply that the N protein has already undergone phase separation to form viable “viral factories” that can interact with DDX3X, facilitating immune evasion and suppression.

### 5.5. DDX3X Is a “Double-Edged Sword” That Mediates Host Antiviral Immunity and Viral Replication

DDX3X is not only an essential mediator of host innate immunity, but also acts as host factors that assist viral replication [532,533]. Therefore, DDX3X is often targeted and hijacked by viruses during infection to evade immune response and promote replication [521,534,535]. SARS-CoV-2 N protein sequesters and potentially binds to DDX3X in order to inhibit host antiviral pathways [530]. The induction of first line IFN immune defense requires the synergistic activation of the type I IFN-β promoter by DDX3X, and TANK-binding kinase 1 (TBK1) and its interaction partner, DDX3X [281,536]. This synergistic effect on IFN induction is mediated by the recruitment of DDX3X into mitochondrial antiviral-signaling protein (MAVS, IPS-1) to promote the scaffolding and aggregation of MAVS into prion-like complexes that can then activate TBK1 and interferon regulatory factor 3 (IRF3) for type I IFN responses [537,538]. LLPS of N protein inhibits both the polyubiquitination and formation of prion-like aggregates in MAVS, effectively suppressing the host innate antiviral response [539]. The prion-like conformational switch of MAVS on the mitochondrial membrane is the lynchpin that propagates antiviral signaling cascades that can inhibit viral infections [540] and is mediated by DDX3X. Nevertheless, in order to hijack DDX3X, viruses including SARS-CoV-2 must first dismantle the assembly of host SGs that are associated with DDX3X.

### 5.6. N Protein Must Phase Separation to Target G3BP1 and Disassemble Stress Granules

Stress granules (SGs) are membraneless organelles assembled as a result of LLPS activated by cellular stress, including viral infections [100,541]. Ras-GTPase-activating protein SH3 domain-binding protein 1 (G3BP1) [542] is the molecular switch that regulates RNA-dependent LLPS of SGs, and its effects on SG LLPS can be tuned by phosphorylation of IDRs in G3BP1 as well as extrinsic binding factors that can strengthen or weaken the SG assembly [543]. G3BP1 promotes SG IFN signaling, enhancing innate antiviral response via positive regulation of RIG-1—an upstream target of MAVS [231,544,545]. Recent biochemical and structural analyses of the interactions between SARS-CoV-2 N protein and G3BP1 revealed that N protein residues 1–25 (N_1–25_) occupies a conserved surface groove of the NTF2-like domain of G3BP1 (G3BP1_NTF2_). The interactions between the N_1–25_ and G3BP1_NTF2_ are enhanced by strong surface complementarity and hydrophobic groove-insertion mechanisms, resulting in the inhibition of SG assembly. However, the underlying mechanism for SG disassembly by SARS-CoV-2 N protein could not be determined [546]. N protein binding to G3BP1 also rewires the G3BP1 mRNA binding profile to suppress host cell stress response [230]. In order to target G3BP1, the SARS-CoV-2 N protein must first undergo LLPS, partitioning into SGs before it can bind and interact with G3BP1 to dismantle assembly of SGs [547].

### 5.7. The Formation of “Viral Factories” by N Protein LLPS Is Tuned by Phosphorylation

Oxidative stress induces the formation of SGs [87], and N protein LLPS induced by oxidative stress in vitro facilitates its partitioning into SGs to sequester G3BP1 [228,513]. Similar to other condensates formed via LLPS, N protein condensates can be tuned by the concentration of RNA where increasing RNA gradient with a fixed protein concentration at 10 μΜ caused N protein to increase viscosity from droplets to gel-like, and, eventually, solid assemblies [270,547], whereas phosphorylation of the serine/arginine (S/R)-rich region in the central IDR of the N protein can tune the viscosity and modulate N protein condensate assembly [278]. Phosphorylation is an ATP-dependent, post-translational modification that can fine-tune molecular interactions of condensate components by inducing nonequilibrium thermodynamic chemical reactions to control the size and number of condensates, acting somewhat like a rheostat that can adjust the dynamics of LLPS during condensate formation [548].

Unphosphorylated N protein facilitates tight association with host mRNAs, and thus, increases the propensity to form gel-like condensates; conversely, phosphorylation of N protein results in the formation of more dynamic, low-viscosity, liquid-like droplets [549]. The EBOV N protein-induced dynamic phosphorylation and dephosphorylation of VP30—the fourth N protein essential for viral transcription—take place in viral inclusion bodies [550,551]. Molecular dynamics simulations revealed that phosphorylation of the phosphate groups at different serine residues in the serine-arginine (SR)-rich domain in SARS-CoV-2 N protein induced the formation of dense salt bridge networks, increasing intra- and intermolecular contacts that impaired contact with RNA derived from SARS-CoV-2 genome, effectively preventing association with nonspecific RNA [132,552]. Thus, the tuning of the physical properties of N protein condensates via phosphorylation can determine whether viral transcription or packaging is favored via hyperphosphorylation (low-viscosity) or hypophosphorylation (high-viscosity), respectively [278,552]. Consequently, high-viscosity, unphosphorylated condensates are more effective at promoting viral packaging—the cytoplasmic compartmentalization of the viral genome—whereas low-viscosity, phosphorylated condensates operate as dynamic “viral factories” to promote viral transcription/replication and host immune evasion [278,549].

## 6. Melatonin Disrupts Formation of “Viral Factories” by Regulating GSK-3 Phosphorylation of N Protein Condensates

N proteins in coronaviruses are important for viral replication because they facilitate template switching that is essential for viral transcription [553] supported by low-viscosity, phosphorylated condensates. The N protein harbors a Gly-rich linker for enhanced phosphorylation by host glycogen synthase kinase (GSK)-3 on the S/R-rich region to facilitate template switching. GSK-3 is a highly conserved and ubiquitously expressed serine/threonine protein kinase with two isoforms—GSK-3⍺ and GSK-3β—in mammals [554]. Both isoforms of the kinase are activated by tyrosine phosphorylation (Tyr^279^, Tyr^216^) [555,556], whereas serine phosphorylation at Ser^21^ and Ser^9^ can inactivate isoforms GSK-3⍺ and GSK-3β, respectively [557,558]. The GSK-3 kinase is implicated in enhancing virus replication, assembly, and release [559,560,561]. As part of the innate antiviral response, GSK-3 acts as a signaling molecule that may be involved in the sensing of nucleic acids of RNA and DNA viruses. It is not only responsible for the rapid activation of type I IFN signaling cascades [562], but also serves as the crux of multiple cell signaling pathways during various stages of viral replication [563]. 

Coronaviruses can hijack host GSK-3 to phosphorylate their N proteins in order to facilitate template switching that enables the smooth transition from discontinuous to continuous transcription, which balances the synthesis of shorter sgmRNAs with full-length gRNAs [167]. Mutations in the Delta and Omicron variants may have allowed increased abundance and hyperphosphorylation of the N protein [564]. Infection by the Delta variant may result in increased viral loads, severity, hospitalization, and ICU admission [565,566,567]. SARS-CoV-2 N protein phosphorylation by GSK-3 at two conserved consensus sites is deemed essential for viral infection and replication; thus, the inhibition of GSK-3 by small molecules is regarded as a viable therapeutic option to reduce infection and potentiate host immune responses [568]. A meta-analysis of clinical data from more than 300,000 COVID-19 patients in three major health systems using a random-effects model revealed a 50% reduction in risks in patients who take lithium which is a direct inhibitor of GSK-3, while in vitro results showed that GSK-3 inhibition effectively impaired viral replication and blocked infection in human lung epithelial cells [569]. Selective screening of a library of GSK-3β inhibitors found a high proportion of compounds that inhibited GSK-3β were also effective in reducing SARS-CoV-2 infection [570].

The activation of GSK-3 in infected cells may be responsible for increased replication and pathophysiology by promoting systemic inflammation, renal dysfunction, and hepatotoxicity via the regulation of cytokine production and cell migration [571,572], as well as the transcriptional regulation of nuclear factor kappa B (NF-κB) [573]. GSK-3 also elevates oxidative stress in infected cells by downregulating the Nrf2 and the Nrf2/antioxidant response element (ARE) pathway [574,575]. GSK-3 directly inhibits nuclear factor erythroid 2-related factor (Nrf2) activation and indirectly inhibits Nrf2 post-induction [576]. The increased oxidative stress from GSK-3 activities may induce the assembly of SGs, but more importantly, the activation of GSK-3 may actually be the elusive, underlying mechanism that is responsible for the disassembly of SGs by SARS-CoV-2 N proteins [546]. While the timely treatment with adequate melatonin essential for attenuating viral infection, replication, and mortality may be dependent upon multipronged strategies employed by melatonin, the inhibition of GSK-3 that can both tune N protein condensate dynamics and rescue stress granule from disassembly by N protein may be one of the most effective tools responsible for the dismantling of the viral replicative machinery.

### 6.1. GSK-3 Phosphorylation of Gle1A Mediates Stress Granule Disassembly via Inhibition of DDX3X

**Figure 2:**
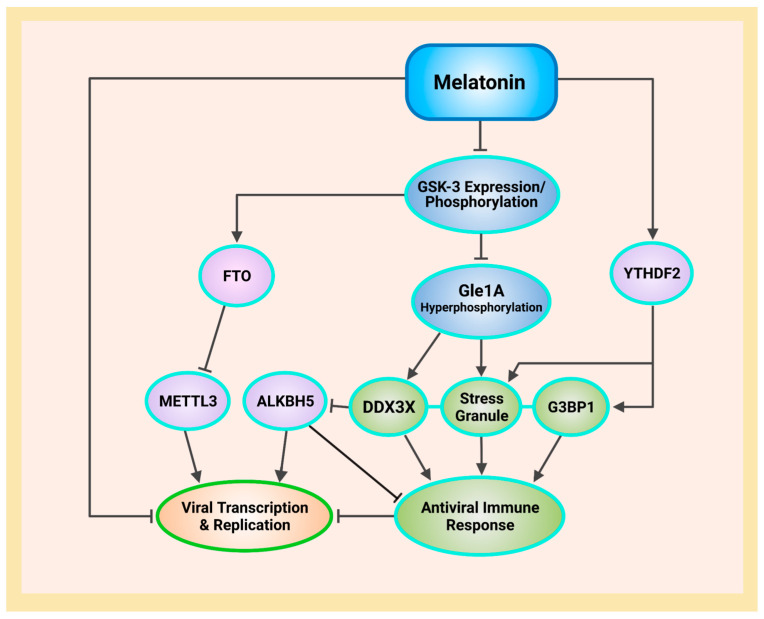
Overview of antioxidant-dependent and -independent mechanisms used by melatonin to regulate m^6^A modifications during SARS-CoV-2 infection and PASC. Melatonin increases the m^6^A “reader” YTHDF2, allowing the proper nucleation of stress granules by G3BP1 to promote antiviral immune response. Melatonin inhibits GSK-3 gene expression and phosphorylation to suppress Gle1A hyperphosphorylation that allows DEAD-box RNA helicase DDX3X to interact with ALKBH5 and stress granules to enhance immune response that reduces viral transcription and replication. The suppression of GSK-3 increases the m^6^A demethylase FTO that “erases” modifications by m^6^A methyltransferase METTL3, decreasing m^6^A levels to suppress viral replication (see Abbreviations for additional acronyms).

The SARS-CoV-2 virus facilitates replication by rewiring cellular metabolism away from OXPHOS within mitochondria in favor of aerobic glycolysis that takes place in the cytoplasm [300,336,337,338,577] to potentially result in a more than a 16-fold reduction in ATP production by OXPHOS [443]. This dramatic shift of ATP production location may serve multiple functions, including the regulation of cytoplasmic N protein condensate dynamics via phosphorylation and the modulation of cytoplasmic SG dynamics. The assembly of SGs and the modulation of their dynamics are regulated by ATP-dependent DEAD-box RNA helicases that reside within the cores of SGs [272]. These RNA helicases function as ATPases to release ATP via hydrolysis in order to maintain the dynamic behavior of liquid-like SG assemblies [541,578]. Many viruses target DDX3X to evade host immune response and facilitate replication [533]. SARS-CoV-2 N protein sequesters DDX3X to promote viral replication [530] by first partitioning into SGs via LLPS, and then disassembling the SGs [513,546] possibly by suppressing interactions between G3BP1 and other SG-related proteins [228]. However, a more direct mechanism may explain how the N protein disassembles SGs and why the timely treatment with melatonin inhibits viral replication and disease progression. The phosphorylation of N protein by GSK-3 not only determines the viscosity and function of condensates formed by N protein LLPS, but GSK-3 can also regulate DDX3X functions to control stress granule assembly and disassembly (Figure 2).

Gene expression pathways respond to stress by exporting critical mRNAs for translation while assembling repressed mRNA into cytoplasmic SGs, the latter being a reversible process [67,579,580,581]. Gle1 is a nucleoporin that regulates mRNA export by activating DEAD-box ATPases [582,583,584] and also modulates translation initiation and termination by tuning DDX3X RNA binding [585]. The human Gle1 gene encodes two distinct isoforms—Gle1A and Gle1B—that modulate SG formation and mRNA export at the nuclear pore complex, respectively [586,587]. When Gle1A is recruited to SGs in the cytoplasm during stress, it becomes a critical modulator of translation that can ultimately affect SG dynamics, assembly, and disassembly by controlling how DDX3X binds to RNA [585,588]. In SGs, DDX3X is the gate-keeper that can either promote translation via RNA binding or suppress translation as a result of excess RNA binding. Gle1A can reduce excess RNA binding by DDX3X (~3-fold reduction), thereby becoming an effective regulator of DDX3X-mediated translation initiation [585]. Upon activation by stress, cytoplasmic Gle1A that is unphosphorylated or minimally phosphorylated at periodically repeating serine/threonine residues in its N terminal IDR, stimulates DDX3X activities to promote SG assembly. However, upon recovery from stress and/or increasing hyperphosphorylation, Gle1A will inhibit DDX3X ATPase activity to promote SG disassembly. GSK-3 is responsible for the phosphorylation of the Ser^88^–Thr^102^ region in Gle1A, altering Gle1A biochemical properties and electrophoretic mobility that facilitate the binding and inhibition of DDX3X ATPase activities to modulate RNA binding. Upon stress, the induction of mitogen-activated protein kinases (MAPKs), including extracellular signal-regulated kinase (ERK) and c-Jun N-terminal kinase (JNK), are required to first prime Gle1A before it can be phosphorylated by GSK-3 [589].

The MAPK family of kinases—ERKs, JNKs, and p38s—control important physiological processes including cell division, transcription, and inflammation, respectively, by phosphorylating and activating each other [590,591]. Melatonin may prevent the disassembly of SGs by Gle1A hyperphosphorylation via the regulation of MAPK kinases. Human osteosarcoma (MG-63) cells treated with 4 mM melatonin for 24 h exhibited significant inhibition of ERK phosphorylation that suppressed signaling and resulted in a marked reduction in cell proliferation [592]. Both in vitro and in vivo work on inflammatory mucin production found melatonin treatment to inhibit phosphorylation of MAPKs including JNK, ERK, and p38. Human epithelial (NCI-H292) cells stimulated with epidermal growth factor (EGF) upregulated expression of mucin mRNA (MUC5AC), production of proinflammatory cytokines, and infiltration of inflammatory cells as a result of enhanced MAPK signaling. Treatment with 400 µM melatonin for 24 h significantly reduced phosphorylation of all MAPKs to reverse all conditions induced by EGF stimulation [593]. Similarly, lung tissues obtained from ovalbumin (OVA)-induced asthma model BALB/c mice supplemented with 15 mg/kg body weight (intraperitoneal injection) prior to OVA challenge all showed reduced phosphorylation of ERK, JNK, and p38, and reduced MUC5AC mRNA expression compared to controls [593]. Spinal cord injury (SCI) model mice displayed edema and loss of myelin in lateral and dorsal funiculi 24 h post-trauma. The loss of motor function characterized by an inflammatory response is mediated by phosphorylation of ERK, JNK, and p38 MAPKs in the spinal cord tissues. Mice treated with melatonin at 50 mg/kg three times within 12 h after laminectomy all showed impressive reduction of SCI-induced functional deficits including improved limb motor functions and effective inhibition of phosphorylation of the ERK, JNK, and p38 MAPKs [594]. To protect SGs from disassembly by Gle1A, melatonin not only inhibits phosphorylation of MAPKs to prevent the priming of GSK-3, but also regulates GSK-3 gene expression and its inactivation.

### 6.2. Melatonin Inhibits GSK-3 Gene Expression and Promotes Phosphorylation to Deactivate GSK-3

Neuro2A cells subjected to okadaic acid (OA) treatment to induce phosphorylation of tau by GSK-3β exhibited elevated ROS and cytotoxicity, resulting in the loss of cell viability of up to 60%. Incubation with 200 µM melatonin for 24 h completely reversed tau-induced cytotoxicity, while at 100 µM concentration, melatonin completely restored cell viability. The addition of only 10 µM melatonin increased the expression of Nrf2 and reduced almost 50% of ROS induced in tau-exposed Neuro2A cells. More importantly, the upregulation of GSK-3β via Tyr^216^ phosphorylation by OA was reversed by the treatment of melatonin. Melatonin effectively reduced the total mRNA expression level of GSK-3β without affecting the phosphorylation of Ser^9^ which can inactivate the kinase [595]. Additionally, in human mesenchymal stem cells, melatonin attenuated adipogenic differentiation by suppressing GSK-3β activities [596], while in mouse osteoblastic MC3T3-E1 cells, melatonin enhanced osteoblastic differentiation and mineralization by inhibiting the phosphorylation of GSK-3β, reversing its negative regulation of the canonical Wnt/β-catenin signaling transduction pathway via phosphorylation and proteasomal degradation of β-catenin [597,598]. Melatonin can reduce ROS and oxidative stress by inhibiting GSK-3 to attenuate the downregulation of the Nrf2 and reactivating the Nrf2/antioxidant response element (ARE) pathway [574,575]. In human epithelial alveolar cells, lipopolysaccharide (LPS)-induced epithelial-mesenchymal transition (EMT) was attenuated by treatment with melatonin in a dose- and time-dependent manner. Treatment with 800 μM melatonin upregulated the phosphorylation of GSK-3β at Ser^9^ to increase the expression of Nrf2 and downstream antioxidant proteins, dramatically reducing malondialdehyde (MDA) levels while increasing antioxidant enzymes [599].

Male Wistar rats subjected to bilateral renal ischemia to induce ischemia/reperfusion (I/R) injury showed increased lipid peroxidation and elevated lactate dehydrogenase (LDH) in plasma compared to controls. Treatment with melatonin (10 mg/kg, i.p.) 30 min before renal clamping markedly reduced lipid peroxidation and LDH levels in plasma, while the phosphorylation of GSK-3β was significantly enhanced via the restoration of AKT phosphorylation in the melatonin-treated group [600]. GSK-3β is a downstream substrate of protein kinase B (AKT), a serine/serine/threonine kinase that phosphorylates GSK-3β under hypoxic stress [601,602]. I/R-induced downregulation of AKT phosphorylation was attenuated by melatonin treatment to enhance inactivation of GSK-3β [600]. Unexpectedly, melatonin was found to modulate the PI3K/AKT pathway, suppressing AKT phosphorylation to activate GSK-3β in SK-MEL-1 and MEL-HO melanoma cells. Compared to respective untreated controls, the addition of 1 mM melatonin dramatically decreased phosphorylation of GSK-3β at 48 h and 72 h, but reduced cell proliferation to ∼50% at 72 h [603]. This seemingly controversial, contradictory behavior may be readily explained by the fact that melatonin is a “smart”, pleiotropic molecule that selects the most appropriate strategy to protect the host organism [604,605,606,607]. The assembly of SGs is one of the major mechanisms used by cancer cells to adapt and survive under inhospitable, toxic microenvironments [608,609], as well as developing resistance to anticancer therapies [610]. Melatonin is known for its anticancer features, acting as a “broad-based metabolic buffer” to tune the cancer microenvironment [415]. Consequently, the inhibition of AKT to activate GSK-3β in melanoma cells may result in the phosphorylation of Gle1A, which subsequently disassembled SGs, explaining the potential mechanism for the reduction of cell viability to ~50% observed at 72 h [603].

Among the seemingly inexhaustible array of sophisticated tactics employed by melatonin to target viral infections, perhaps the ability of melatonin to regulate epitranscriptomic and epigenetic modifications mediated by viruses, including SARS-CoV-2, is the most influential, with the broadest, most profound implications for PASC development post-infection.

## 7. Melatonin Regulates SARS-CoV-2-Mediated Crosstalk between the Epitranscriptome and Transcriptome via m^6^A Modifications and LINE1 Suppression

The reversible chemical modification of mRNA is a potent modulator of transcription and translation in the epigenetic regulation of genomic DNA [611,612]. The important crosstalk between epitranscriptomic and transcriptomic modifications underlies the success of post-transcriptional regulation of gene expression during growth and development, as well as response to exogenous and endogenous stress [244,613,614]. The SARS-CoV-2 virus can remodel one-third of the RNA-bound proteome (RBPome), upregulating and downregulating more than 300 RNA-binding proteins (RBPs) [158] to exert profound influences on the host and viral epitranscriptome [615] that not only affect disease progression, but also post-infection development of PASC. To evade host immune detection, the SARS-CoV-2 virus can methylate the 5′-end of virally encoded mRNAs to mimic cellular mRNAs [616]. Furthermore, COVID-19 patients exhibited marked alteration in circulating platelet gene-expression profile and distinct changes in gene expression in pathways involving protein ubiquitination, antigen presentation, and mitochondrial dysfunction [617]. Olfactory biopsies from hyposmic PASC patients with persistent loss of smell from olfactory dysfunction at least 4 months post-infection revealed changes in sententacular cell and olfactory sensory neuron phenotypes, including a reduction in relative cell number and expression of signaling intermediates [618].

Longitudinal multi-omics analyses in peripheral blood samples from hospitalized COVID-19 patients discovered dynamic changes in circulating blood cells, where fatal outcomes were associated with specific cell-type expression signatures, in addition to epigenetic changes in gene expression involving genome-wide hypomethylation when compared to healthy controls [619]. An analysis of peripheral blood samples from young adult COVID-19 patients (average age 35.7 years), via RNA and whole--genome bisulfite sequencing three months after recovery, found dramatic alterations in both the expression and DNA methylation of genes and transposable elements (TEs), with a total of 639 misregulated genes and 18,516 differentially methylated regions (DMRs). More importantly, 13,233 DMRs were identified within the TE loci, with 36.48% allocated to LINE, while the significant level of aberrant activation of TEs corresponded with disease severity, as TEs with altered DNA methylation may regulate adjacent gene expression [620]. Osteoporosis is often associated with alterations in DNA methylation profiles in cancellous bones [621]. Disturbances in bone metabolism in recovered COVID-19 patients are associated with dysregulation in osteoclastic activities with increased bone resorption mediated by an imbalance in the RANKL/RANK/OPG axis [622,623]. Syrian hamsters infected by SARS-CoV-2 all exhibited significant multifocal loss of cancellous bones as a result of elevated osteoclastogenesis. Compared to mock controls, infected hamsters exhibited a dramatic three-fold increase in the expression of the pro-osteoclastic receptor activator of nuclear factor-kappa B ligand (RANKL), while the expression of osteoprotegerin (OPG), which inhibits osteoclastogenesis and bone resorption, was correspondingly downregulated [624]. Thus, the transcriptomic and epigenomic changes from SARS-CoV-2 infections may critically define and drive the development of PASC during post-infection recovery. 

### 7.1. SARS-CoV-2 Derepression of LINE1 May Induce Genomic Instability That Exacerbates Disease Severity and Prolongs Recovery

The successful strategies employed by viruses for infection and replication depend upon the effective control and utilization of host cellular metabolism and transcriptional processes that often result in transcriptional changes for both host and virus. The SARS-CoV and SARS-CoV-2 viruses facilitate infection and replication, inhibiting immune responses by targeting the host transcriptome, suppressing host gene expression by degrading host mRNA, preventing IFN-β mRNA accumulation [56], and disrupting host mRNA splicing and protein translation to suppress IFN signaling pathways [55]. A multicenter observational study of 234 COVID-19 patients with acute respiratory illnesses (ARIs) comparing transcriptional signatures between SARS-CoV-2 and other respiratory viruses found attenuated activation of innate immune responses including toll-like receptor, interleukin, and chemokine signaling [625]. An analysis of transcriptional profiles of bronchoalveolar lavage fluid (BALF) from severe COVID-19 patients exhibiting pneumonia revealed profound changes in mRNA levels of encoding proteins that regulate coagulation, fibrinolysis, and inflammation, where a reduction of 22-fold and 33-fold in transcripts encoding thrombomodulin and endothelial protein C receptor (EPCR), respectively, compared to controls, were detected [626]. The dysregulation of repetitive elements, including LINE1, can easily alter gene expression and cause changes in the cellular transcriptome due to proximity of location. The FANTOM4 projected reported that between 6 to 30% of cap-selected mouse and human RNA transcripts are initiated within repetitive elements [627]. Human LINE1 antisense promoter driven transcripts are transcribed in a variety of cell types, comprising ~4% of all human genes [628]. The activation of LINE1 (L1) and L1 antisense promoter (ASP) that drive mRNA production and L1-gene chimeric RNA production, respectively, are associated with a wide range of pathologies, including cancer and genetic diseases [629,630,631,632,633,634].

The insertion of DNA sequences by TEs accounts for ~45% of the human genome. DNA transposons are no longer active, while RNA transposons (retrotransposons, retrotransposable elements, RTEs) have remained active in the genomes of all eukaryotes and many prokaryotes over the past 80 million years, reversibly altering the expression of other genes and serving as a rich source of genetic diversity [632,635,636]. Due to the high mobility of TEs, they were known as “jumping genes” after their discovery by Barbara McClintoch in 1948 [637,638]. Representing 17% of the human genome, long interspersed nuclear element 1 (LINE1, L1) retrotransposons are a large family of autonomous mobile, repeated deoxyribonucleic acid (DNA) elements capable of generating genomic instability and DNA damage [639,640,641,642] that often result in diseases such as cancer [643]. Even though the mobility of L1 is highly repressed in most somatic cells, it can escape repression and is actively transcribed in many somatic cells [644], including quiescent, nondividing, differentiated primary somatic cells [645]. The derepression and associated hypomethylation in cancer cells are regarded as effective biomarkers of neoplasia [646]. Derepressed L1 transposition is consistently correlated with p53 mutation and replication stress that induces copy number alterations [629].

The full-length LINE1 comprises a 5′- and a 3′-untranslatable region (5′, 3’-UTR) [647], and two open reading frames—ORF1 and ORF2—that encode RNA binding protein ORF1p and ORF2p, respectively. ORF1p is an RNA binding protein, while ORF2p has an endonuclease domain and a central reverse transcriptase (RT) domain that can polymerize hundreds of nucleotides per template binding event [648,649]. In tumor cells, ORF2p RT can sequester RNA for reverse transcription, forming RNA:DNA hybrid molecules that can impact global cell transcription on the epigenetic level [650]. Furthermore, the insertion of L1 sequences on transcripts can dramatically decrease RNA production of endogenous genes, qualitatively and quantitatively, leading to dramatic alterations in the transcriptome [651].

#### 7.1.1. Can SARS-CoV-2 Be Reverse-Transcribed to Form Viral-Host Chimeric Transcripts?

Retroviruses replicate by integrating viral RNA by reverse transcription into the host genome [652]. A nonretroviral RNA virus, the lymphocytic choriomeningitis virus (LMCV) recombines with retrotransposons to invade the host genome [653]. Even though coronavirus RNAs are not supposed to reverse transcribe and integrate into host DNA, recent works by Yin et al. found infection by SARS-CoV-2 and other human coronaviruses not only upregulated the expression of RTEs in various cell types, but also led to the formation of chimeric transcripts with LINE1 for potential insertion in to the host genome [654], while Zhang et al. found evidence that a high percentage of all viral transcripts in certain patient tissues were derived from viral-host integrated sequences expressed as chimeric transcripts. Furthermore, Zhang and co-workers were able to detect and clone DNA copies of the N protein, implying that SARS-CoV-2 RNAs can be reverse-transcribed by LINE1 and integrated into the host cell genomes [655]. The works on SARS-CoV-2 reverse transcription sparked a flurry of animated debates in the scientific community, where various counter perspectives were presented, including the possibility that human SARS-CoV-2 chimeric reads were formed during RNA sequencing (RNA-seq) library construction [656], the lack of reproducibility [657], and the hypothesis being questionable [658]. Even though a consensus on this controversial topic may not be reached easily, there is ample evidence that show SARS-CoV-2 infections can derepress and activate LINE1 to cause genomic instability and host gene misregulation, potentially increasing disease severity and prolonging recovery.

#### 7.1.2. LINE1 Derepression and Global Hypomethylation May Be Associated with SARS-CoV-2-Mediated Pathologies

PASC patients in the absence of persistent infections often exhibit symptoms resembling persistent viral infections with aberrant activation of innate immune signaling pathways resulting in a multitude of pathologies associated with chronic inflammation [659]. An integrated analysis of blood samples from convalescent COVID-19 patients at 12, 16, and 24 weeks (±14 days) post-infection (pi), examining immune responses at a transcriptional level found persistent alterations in immune cell populations up to 24 weeks pi, while severe disturbances in gene expression were identified in whole blood RNA sequencing analyses at up to 6 months pi [660]. LINE1 modulates the immune microenvironment during derepression where activated L1 retrotransposons can elicit strong innate immune responses, and induce autoimmunity and inflammation [661]. Differential gene expression analysis identified an upregulation and downregulation of 738 and 230 genes, respectively, in COVID-19 convalescent patients at 12 weeks pi compared to healthy controls. The innate immune system pathway was substantially enriched among the genes that were upregulated [660]. An analysis of DNA methylation of blood samples collected from 413 COVID-19 patients and 232 healthy subjects revealed that accelerated epigenetic aging is associated with the development of not only severe COVID-19, but also may contribute to the development of PASC [662].

A massive retrospective cohort study that examined data from the Veterans Health Administration involving over 2.7 million veterans found a higher risk of incident diabetes in male veterans with positive SARS-CoV-2 test results than those with negative results; moreover, hospitalized male subjects were associated with higher risk of diabetes at 120 days and at the end of follow-up [663]. A global registry for patients with COVID-19-associated diabetes was established in 2020 to facilitate the study of new-onset diabetes in COVID-19 patients [664]. Furthermore, individuals infected by SARS-CoV-2 under the age of 18 had increased risk for new-onset diabetes >30 days after infection than those without COVID-19 [665]. Currently, there is a lack of understanding on the precise mechanism that triggers new-onset diabetes in PASC [666]. It is perhaps not a coincidence that lower LINE1 methylation is correlated with a greater risk for metabolic syndrome and related phenotypes [667], and can be used as a biomarker for weight loss and total antioxidant capacity in obese subjects [668]. Analysis of data collected from a prospective cohort intervention study and a control group revealed reduced LINE1 methylation levels were directly associated with carbohydrate metabolism disorders, worsening of metabolic status, and risk of developing metabolic disorders including type 2 diabetes. More importantly, the association of these higher risk factors with lower LINE1 methylation were independent of other classic risk factors, including gender, physical activity, body mass index (BMI), and especially age [669]. The demethylation of RTEs is often associated with the aging process [670].

During aging, epigenetic changes that affect genome stability and regulation are often characterized by the establishment of genome-wide global hypomethylation and promoter-specific hypermethylation [671,672]. The loss of genome-wide methylation, or the establishment of global hypomethylation, is an epigenetic event often associated not only with aging, but also many types of cancers [673] and a growing list of other pathologies. RTE hypomelation is associated with transcriptional derepression of LINE1 [674]. During replicative senescence, L1 is derepressed and becomes active in somatic retrotransposition to negatively impact longevity [675,676]. Consequently, LINE1 derepression in senescent cells is regarded as a hallmark of aging as a result of heightened elevation of interferon and sterile inflammation from L1 activation [677]. Due to the large presence of TEs in the genome, the methylation status LINE1 is, therefore, widely used as a surrogate in the research of various diseases to accurately identify DNA methylation status often referred to as “global” methylation [678,679,680,681,682,683].

Results from RNA and whole-genome bisulfite sequencing of blood samples from recovered COVID-19 patients (average age 35.7 years) revealed a total of 13,233 DMRs within the TE loci, where LINE DMRs comprised 38.23% of the total DMRS [620] and longitudinal multi-omics analyses in peripheral blood samples from hospitalized COVID-19 patients showed genome-wide hypomethylation when compared to healthy controls [619]. The global hypomethylation of LINE1 may be responsible for the development of various PASC pathophysiological where major manifestations in addition to immunoregulatory dysfunction [684] include cardiovascular autonomic dysfunction [350,354] and neurological dysfunction [685]. A prospective, observational evaluation of PASC patients at a mean of 5.8 ± 3.5 months after symptom onset, employing head-up tilt table (HUTT) testing to determine orthostatic intolerance, discovered orthostatic intolerance suggestive of autonomic dysfunction in nearly all subjects who complained of reduced exertional tolerance, and onset of palpitations and tachycardia with minimal activity level [350]. Furthermore, symptoms including fatigue, chest pain, dyspnea, and “brain fog”, in addition to cardiovascular autonomic dysfunction, are common among PASC patients [354,686].

Myocardial injury is an important pathogenic feature of COVID-19, where cardiovascular histopathology findings are reported in up to 48% of patients; moreover, cardiovascular magnetic resonance abnormalities are detected in 26% to 60% of recovered hospitalized patients [687,688,689,690]. The frequent presence of elevated high-sensitive troponin I (hs-TnI) in COVID-19 patients is significantly associated with higher rates of cardiac complications [691]. Even though immune-mediated inflammation and hypercoagulability [692], the dysregulation in the CD147-cyclophilin pathway [693], as well as other potential direct and indirect mechanisms have been proposed [694], the exact causal mechanisms for myocardial injury in COVID-19 still await further elucidation [695]. Whether the SARS-CoV-2 virus directly mediates cardiac injury also remains controversial. An examination of cardiac tissues from 39 consecutive autopsy cases of COVID-19 subjects revealed the presence of the virus in 61.5% (24/39) of cases, while viral load above 1000 copies per μg RNA was detected in 41% (16/39) of subjects [696]. Conversely, although autopsy results of 40 patients deceased with severe SARS-CoV-2 all exhibited evidence of both chronic and acute myocardial damage, viral genome and spike protein were noticeably absent in the cardiomyocytes of the only patient with myocarditis, while patients with known viral persistence in the lungs and no signs of myocardial inflammation presented a negligible presence of viral particles in their cardiomyocytes [697]. Interestingly, LINE1 hypomethylation is associated with increased cardiovascular disease risk in different ethnicities [698,699], as well as increased risk for myocardial infarction [700]. Myocardial infarction and myocardial injury are directly associated with autonomic dysfunction [701,702].

In infants, LINE1 hypomethylation is associated with elevated risk for tetralogy of Fallot (TOF), which is a congenital defect caused by improper development of the right side of the heart, usually characterized by right ventricular (RV) hypertrophy [703]. Patients with pulmonary arterial hypertension (PAH) with RV hypertrophy exhibited significant impairment in autonomic balance compared to healthy controls [704]. Interestingly, complete echocardiographic evaluation of COVID-19 patients conducted within 24 h of admission found RV dilatation and dysfunction in 39% of patients, while the most common echocardiographic abnormality at follow-up of patients (20%) who experienced clinical deterioration was RV function deterioration [705]. Additionally, 78% (78/100) of recovered patients showed cardiac involvement in their MRIs executed 71 days (average) pi, while 60% showed persistent myocardial inflammation [706].

#### 7.1.3. LINE1 Derepression and Global Hypomethylation Are Induced by Mitochondrial Dysfunction

Global hypomethylation is associated with LINE1 derepression [674]. Generally repressed in somatic cells, L1s are activated in the embryo, and the de novo retrotransposition events in the germline by highly polymorphic L1s may contribute to heritable genetic variations and mutations. Since the reactivation of L1s in the germline and the loss of global DNA methylation threatens the stability of the germline genome, L1s are usually silenced by adaptive mechanisms in the germline [707,708], whereas derepression causing global hypomethylation of LINE1 is associated with many types of cancers [679,709,710,711]. The only known somatic tissue in humans where L1s are derepressed throughout the entire life cycle is the human brain [712]. L1s can make new somatic insertions and mobilize DNA copies via copy-and-paste duplication mechanisms [637]. It is estimated that there are ~13.7 new somatic L1 insertions per hippocampal neuron, and 6.5 insertions per glial cell [713], with implications of potential relevance during neuronal differentiation and the generation of genomic plasticity [714], while having the capacity to influence not only neurogenesis [715], but also normal and abnormal neurobiological processes [716]. Inevitably, many neurological disorders are associated with complex, aberrant L1 activities [635], including Rett Syndrome (RTT) [717], Aicardi-Goutières syndrome (AGS) [718,719], ataxia telangiectasia (AT) [720], autism spectrum disorder (ASD) [721,722], schizophrenia [723], amyotrophic lateral sclerosis (ALS) [724], and neurodegenerative disorders [725,726].

SARS-CoV-2 infections can cause mitochondrial dysfunction via multiple mechanisms, including elevated ROS, membrane depolarization, and alterations in mtDNA gene expression and copy number [298,336] (Section 5.1, Section 5.2 and Section 5.3). LINE1 can be mobilized in neurons by distressed mitochondria where mild inhibition of mitochondrial complex I activity increased free radical production to activate the mobilization of LINE1 in male C57Bl/6J mice in vivo and human dopaminergic LUHMES cells in vitro. The resultant global demethylation may induce epigenetic alterations in the transcription of genes that encode mitochondrially imported proteins [727]. It is, therefore, entirely conceivable that mitochondrial dysfunction from viral persistence in PASC patients may be responsible for common, major manifestations, such as immune dysregulation, cardiovascular autonomic dysfunction, and neurological dysfunction via mobilization of LINE1 and subsequent epigenetic alterations. Melatonin is an ancient molecule that not only can regulate viral phase separation to inhibit infection and replication, but also suppress LINE1 derepression.

### 7.2. Melatonin Suppresses LINE1 Derepression via Antioxidant-Dependent and -Independent Mechanisms

Oxidative stress is widely accepted as the key player contributing to the pathogenesis, severity, and mortality of COVID-19 patients [728]. The only difference in clinical parameters between critically ill patients who recovered or died is the level of 4-HNE-protein adducts [397], implying a high level of lipid peroxidation from uncontrolled oxidative stress potentially induced by various mechanisms during viral infection, including viroporin-mediated membrane depolarization and mPTP opening (Section 5.2). In human bladder cancer tissues, the expression of the RNA-binding LINE1 ORF1 protein was elevated by increased 4-HNE as well as H_2_O_2_-induced oxidative stress in various cell cultures [729], while a comparison between peripheral blood cells, urinary exfoliated cells, and cancerous tissues collected from healthy controls and bladder cancer patients found marked differences in LINE1 hypomethylation patterns between the two groups. However, both healthy and cancerous specimens exhibited a distinct, dose-dependent, positive correlation between LINE1 hypomethylation and the level of oxidative stress as represented by urinary total antioxidant status (TAS) and plasma protein carbonyl content [730]. During infections, increased expression of oxidative stress genes supports pro-inflammatory immune responses [731,732]. In silico analyses evaluating the expression of 125 oxidative stress genes from publicly available transcriptomic datasets of COVID-19 patients found a significant upregulation of seven oxidative stress genes in severe versus nonsevere COVID-19, while important antioxidant genes were downregulated at critical disease stages. Furthermore, saliva and blood samples revealed a significant increase in myeloperoxidase and calprotectin—oxidative stress genes responsible for inflammatory host defense that were detected by in silico analyses—in severe patients compared to asymptomatic COVID-19 patients [733,734,735].

#### 7.2.1. Oxidative Stress Activates LINE1 ORF1 Proteins to Associate with Stress Granules

The derepression of LINE1 triggered by oxidative stress may be one of the evolutionary adaptive responses, where living organisms increase genotypic and phenotypic variations during exogenous and/or endogenous stress [676,736], in addition to the formation of reversible SGs and other stress-induced, phase-separated MLOs [69]. In plants, RTEs are also activated upon stress to support somaclonal variation, altering gene expressions that may provide evolutionary advantages [737,738]. What remains unclear is whether the production of L1 genetic variations in humans exerts a toll in the form of DNA damage [642]. However, recent works revealed the important role of L1 genotoxicity serving as quality control for genome integrity in fetal oogenesis that may promote genetic diversity and adaptation to stress in mammals [739]. BE(2)C neuroblastoma cells—representative of sympathetic neuron embryonic precursors—treated with H_2_O_2_ to induce oxidative stress generated a two-fold increase in LINE1 ORF1p mRNA transcripts [740]. Mouse embryonic fibroblasts incubated with H_2_O_2_ elicited the assembly of SGs [86], and not unexpectedly, LINE1 ORF1 proteins are targeted to SGs, colocalizing with markers of cytoplasmic SGs in both stressed and unstressed cells, implying that SG formation and the tight association with LINE1 ORF1ps are intended to control retrotransposition activities and genetic alterations, including potential DNA damage [741,742]. Thus, by preventing SG disassembly via inhibition and deactivation of GSK-3 (Section 6.2, Figure 2), melatonin may suppress aberrant LINE1 activities as a result of SARS-CoV-2 targeted disruptions of host SGs. Most importantly, melatonin can inhibit LINE1 derepression in an antioxidant-dependent and -independent manner.

#### 7.2.2. Melatonin May Inhibit LINE1 Expression and Derepression via Regulation of ORF1 Protein Phase Separation

An in situ perfusion of human prostate cancer-derived tumors established in nude male rats with human blood collected from healthy male donors at different times during the circadian cycle revealed that only blood rich in melatonin could suppress endogenous L1 mRNA expression. In 2014, deHaro and co-workers reported the discovery that blood collected from donors at night after exposure to bright light for 1 h resulted in a marked suppression of endogenous melatonin production; moreover, perfusion of tumors using blood exposed to bright light at night failed to reduce L1 mRNA in the same manner as the melatonin-rich blood collected at night without light exposure from the same donors [743,744]. Conversely, adding exogenous melatonin to melatonin-deficient blood during in situ perfusions suppressed L1 mRNA in the same manner as nighttime melatonin rich-blood. Furthermore, the overexpression of melatonin receptor 1 (MT1) also reduced L1 mRNA and ORF1p in cultured cells [743]. Since ORF1p phosphorylation is required for L1 retrotransposition [745], and the phosphorylation of ORF1p sites target proline-directed kinases (PDPKs), including MAPKs and GSK-3 [746], it is not inconceivable that inhibition of MAPKs and GSK-3 by melatonin can interfere with ORF1p phosphorylation to suppress L1 retrotransposition. However, it is highly probable that melatonin may inhibit L1 retrotransposition via the regulation of ORF1p phase separation and other antioxidant-dependent and -independent mechanisms.

#### 7.2.3. ORF1p Phase Separation Formation of Dynamic Condensates Is a Requisite for L1 Retrotransposition

Both full-length and truncated ORF1p that contain the intrinsically disordered N-terminal domain (ORF_1–53_) and coiled-coil domain (ORF1_1–152_) are capable of robust phase separation that form dynamic cytoplasmic membraneless condensates [747,748]. Even though the disordered N-terminal domain promotes LLPS, phase separation of ORF1p is dependent upon the interactions between the N-terminal and coiled-coil domains; furthermore, LLPS of ORF1p can be inhibited by high salt concentration [747], where no condensate formation was observed above 300 mM potassium chloride (KCl) [748]. Decreasing the ratio of ORF1p to RNA changes the viscosity and surface tension of condensates, slowing droplet fusion kinetics, and even alters the physical properties of L1 condensates [748]. More importantly, ORF1p phase separation may be essential for L1 retrotransposition, as mutations that prevented ORF1 condensate formation also suppressed retrotransposition activities [748]. Therefore, inhibition of ORF1p phase separation may be one of the mechanisms employed by melatonin to suppress L1 derepression.

#### 7.2.4. Melatonin Enhances Complex I Functions, Reduces Oxidative Stress, and Regulates DNA Damage Response Elements to Restrain L1 Retrotransposition

L1 derepression in neurons can be activated by distressed mitochondria exhibiting inhibition of complex I activities [727]. Early in vivo and in vitro works found melatonin to be extremely effective in counteracting oxidative stress, protecting and enhancing mitochondrial OXPHOS activities to elevate ATP production in mitochondria. Male Wistar rats treated with ruthenium red (60 mg/kg bw)—an inhibitor of mitochondrial OXPHOS [749]—exhibited extensive oxidative stress and damage in their liver and brain mitochondria. Administration of melatonin at 10 mg/kg (i.p.) 10 min before ruthenium red treatment not only rescued, but increased activities of complex I in a time-dependent manner, where maximal responses observed at 30 and 60 min returned to control levels after 120 and 180 min in liver and brain mitochondria, respectively [750]. Furthermore, in vitro work showed that melatonin rescued cyanide-induced inhibition of ATP production in rat brain and liver mitochondria by counteracting the deactivation of complex IV by potassium cyanide (CN) in a dose-dependent manner. The addition of 1 and 10 nM melatonin significantly enhanced complex I activity in cyanide-treated mitochondria in liver and brain, respectively. Whereas the addition of 100 nm melatonin not only counteracted complex IV toxicity induced by CN, but also increased complex IV activity to 50% above control levels to generate an impressive 46% increase in ATP production [265]. In neurons, ruthenium red inhibits mitochondrial OXPHOS by K^+^ depolarization that maximally elevates Ca^2+^ levels [751], in a manner not dissimilar to viroporin ion channel activities that can cause mitochondrial distress. The ability of melatonin to rescue mitochondria from ruthenium red-induced ionic imbalance and cyanide-induced oxidative stress [752] in mitochondria shows melatonin can be an effective suppressor of L1 derepression. Furthermore, melatonin may be able to maintain BRCA1 gene expression and regulate S phase DNA damage repair to restrain L1 retrotransposition.

A sophisticated spatio-temporal analysis of host transcriptomics from autopsy samples of cardiac tissues obtained from COVID-19 patients revealed that, although absent in cardiac tissues, SARS-CoV-2 can cause extensive DNA damage and the consequential upregulation of genes associated with DNA damage repair. Whereas the downregulation of gene clusters associated in mitochondrial function and metabolic regulation in cardiac tissues may be responsible for the evasion of mitochondria-mediated innate immunity [753]. The SARS-CoV-2 virus spike protein may cause dysregulation of DNA damage response by preventing the recruitment of key DNA repair proteins, including the E3 ubiquitin ligase Breast Cancer 1 (BRCA1) to targeted damage sites [754,755]. BRCA1 directly affects L1 retrotransposition frequency and L1 structure via competition during the important cell cycle checkpoints S/G2 phase [756,757]. L1 is biased towards the DNA synthesis S phase of the cell cycle where genetic replication is the most vulnerable [756,758], and ORF2p is responsible for binding essential S phase replication fork components PCNA and MCM [756,759]. Therefore, overexpression of L1 can result in the overaccumulation of cells stalled in the early S phase, implying that L1 not only can enhance but also relies on replication fork stalling for retrotransposition [756]. BRCA1 may also suppress L1 by regulating L1 ORF2p translation via mRNA binding between ORF1p and ORF2p to impede translation and affect ORF2 levels [756]. Even though melatonin is known to downregulate the expression of BRCA1 genes that were elevated by estradiol stimulation in several breast cancer cell lines [760,761], not surprisingly, melatonin is associated with the nighttime elevation of BRCA1.

BRCA1 gene expression in lymphocytes of shift workers exhibited reduced amplitude compared to healthy daytime workers. Furthermore, BRCA1 expression in healthy day workers peaked at night, whereas BRCA1 levels for shift workers were the lowest at night [762]. Even though the correlation between altered BRCA1 expression and melatonin levels in shift workers was not determined, the increased light exposure at night naturally suppresses melatonin production [763], altering BRCA1 gene expression [764,765]. Nevertheless, the role of melatonin as a “smart”, “pleiotropic” molecule that modulates DNA damage response and repair pathways has been extensively reported and reviewed [766,767,768,769,770]. In breast cancer cells, pretreatment with melatonin from 1 nM to 1 mM, seven days before irradiation, resulted not only in a significant decline of cancer cell proliferation compared to radiation alone, but also induced a significant decrease in proportion of cells in the S phase and a simultaneous increase in proportion of cells in the G1 phase [771]. In addition to the reduction of cells in the S phase and elevation of BRCA1 as part of an impressive array of strategies to contain L1 derepression, melatonin also dynamically deploys m^6^A epitranscriptomic modification to regulate phase separation not only to restrain L1 derepression, but also suppress SARS-CoV-2 viral replication (Figure 1).

### 7.3. m^6^A Modifications Regulate SARS-CoV-2-Mediated LINE1 Derepression

LINE1 is the driving force behind both genome diversity and genome instability [772,773]. Hosts including humans rely upon an intricate balance of m^6^A modifications to safeguard genome integrity. Thousands of m^6^A-marked intronic L1s (MILs) discovered in a variety of fetal tissues can block the transcription of long genes and impede host gene expression resulting in disease; conversely, the host uses effective countermeasures including the nuclear matrix RNA binding-protein SAFB [774]—a novel m^6^A reader complex—to bind and reduce MILs and protect host gene transcription processes [775]. In general, m^6^A positively regulates the expression of autonomous L1s and facilitates L1 retrotransposition by promoting the docking of eukaryotic initiation factor 3 (eIF3) on L1 5′ UTR to generate retrotransposition-competent L1 RNPs, whereas the m^6^A “eraser” ALKBH5 suppresses retrotransposition [776]. Therefore, the depletion of SAFB can significantly elevate L1 retrotransposition activity, but knockdown of “writers” and “readers” will impede the process. Furthermore, the depletion of m^6^A “writers” METTL3, METTL14, and ZC3H13 or “reader” YTHDC1 will reduce the levels of the evolutionarily young intronic L1s that are marked by m^6^A [775,777].

RNA regulates phase separation of condensates by contributing to multivalency through nonspecific negative charges [242,243]; moreover, m^6^A modifications add another layer of control to RNA-mediated phase separation by altering the charge, conformation, and anchoring of RNA-binding proteins (RBPs). m^6^A modifications act as “beacons” to attract “readers”, such as YTH domain proteins that binds m^6^A to modify interactions between RNAs and RNAs and proteins via RNA splicing, folding, and protein translation [246,778]. The depletion of the m^6^A “reader” YTHDC1 in mouse embryonic stem cells (ESCs) resulted in the dysfunction in RNA recruitment, preventing the proper formation of LINE1-scaffold complexes vital in the maintenance of ESC self-renewal [779]. In order to promote viral gene expression, both DNA and RNA viruses have successfully evolved mechanisms to take full advantage of host epitranscriptomic modifications that may positively regulate mRNA translation to maximize viral gene expression [245]. In essence, the regulation of host transcriptome and epitranscriptome by viruses including SARS-CoV-2 ultimately converges on m^6^A modifications [780].

### 7.4. Viral Epitranscriptomics: The Hijacking of Host m^6^A for Viral Infection and Replication

**Figure 3:**
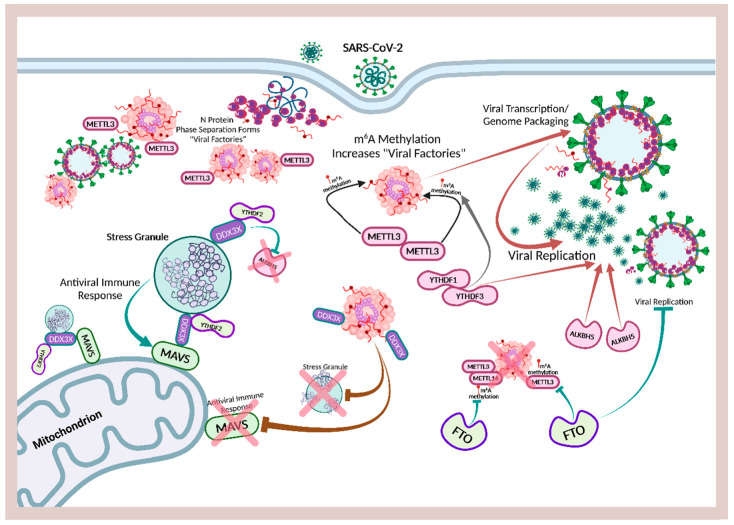
Overview of m^6^A modification effects on SARS-CoV-2 viral replication.

The epitranscriptome is a collection of ~163 post-transcriptional chemical modifications of eukaryotic messenger RNAs (mRNAs) [781,782] responsible not only for the regulation of fundamental biological processes, but also gene expression and regulation at the RNA level [783,784,785,786]. Reversible epitranscriptomic changes can fine-tune gene expression to modulate stress responses and developmental processes [244]. *N*^6^-methyladenosine (m^6^A)—the most abundant, dynamic, reversible modification that transfers a methyl group to the sixth position of the purine ring in RNA adenosine—has been found in the genomes of RNA viruses and is responsible for both viral inhibition and replication in host cells [247,787,788]. The replication of SARS-CoV-2—similar to other positive-strand, eukaryotic RNA viruses—transpires exclusively in the cytoplasm of host cells [789], and m^6^A covalent editing events in the cytoplasm can alter viral gene expression to regulate infection, replication, and pathogenesis [790,791,792] (Figure 3).

During infection, the human respiratory syncytial virus (RSV) can alter host RNA m^6^A distribution, where more than 7000 alterations in host gene expression in human lung carcinoma epithelial A549 cells were partially attributed to m^6^A modifications. Furthermore, m^6^A “writers” enhanced RSV replication and pathogenesis, and knockdown of methyltransferases METTL3 and METTL14 decreased RSV replication; whereas knocking down demethylases, on the contrary, increased viral gene expression and replication. In the same manner, overexpression of m^6^A “erasers” FTO and ALKBH5 produced a remarkable reduction in expression of RSV F and G replication proteins by 80-and 20-fold, respectively [793]. Similarly, the influenza A virus (IAV) expresses m^6^A-modified RNAs that control viral expression and pathogenicity, where the in vitro replication of IAV in A549 cells is inhibited by mutational inactivation of the METTL3 m^6^A “writer” enzyme. Conversely, IAV gene expression, replication, and production of infectious particles are enhanced by the ectopic overexpression of the YTHDF2 m^6^A reader enzyme [794]. Studies employing a combination strategy of gene knockdown, knockout and overexpression found m^6^A residues to enhance replication of IAV [794]. Overexpression of the “reader” YTHDF2 or the knockdown of the ALKBH5 m^6^A “eraser” enzyme promoted HIV-1 gene expression and replication, whereas the knockdown of METTL3 or YTHDF2 both inhibited HIV-1 gene expression [795,796]. The ZIKV, however, responds differently to host m^6^A modifications where knockdown of “writers” METTL3 and METTL14 increased ZIKV production, whereas silencing demethylases and “reader” YTHDF increased replication of the Zika virus [797].

M^6^A methyltransferases (“writer”) METTL3 facilitates SARS-CoV-2 nucleocapsid (N) protein phase separation to form viral factories. M^6^A modification is enhanced and promoted by “readers” from the YTH domain proteins YTHDF1 and YTHDF3. N protein phase separation disassembles stress granules and hijacks DEAD-box RNA helicase DDX3X to allow m^6^A demethylase (“eraser”) ALKBH5 to remove m^6^A modifications in antiviral transcripts, suppressing innate immunity to increase viral replication. Conversely, m^6^A “eraser” FTO can inhibit viral replication by removing modifications by “writers”, effectively reducing formation of viral factories responsible for viral transcription and genome packaging. M^6^A “reader” YTHDF2 facilitates the assembly and nucleation of stress granules, ensuring DDX3X is allocated to support antiviral immune responses, including MAVS in mitochondria. 

### 7.5. Is m^6^A a Positive or Negative Regulator of SARS-CoV-2 Replication?

The RNA modification of eukaryotic RNAs by m^6^A is dynamic and reversible. Methylation by “writers”, including METTL3 and METTL14 of mRNAs, transfer RNAs (tRNAs), ribosomal RNAs (rRNAs), and long noncoding RNAs (lncRNAs), can be reversed or “erased” by demethylases such as FTO and ALKBH5 that remove the m^6^A from RNAs [798,799,800,801]. The SARS-CoV-2 virus mediates virus–host interactions to enhance replication by modifying m^6^A and increasing the host m^6^A methylome upon infection. In 2021, Liu et al. demonstrated for the firsts time that the SARS-CoV-2 negative-sense RNA intermediates, in addition to the positive-sense genome, were also subject to m^6^A epitranscriptomic regulation in infected cells where dynamic changes to both host and viral RNA m^6^A methylome were detected [802]. The SARS-CoV-2 virus can modulate methylation motifs in mRNAs and translocate the m^6^A “writer” METTL14 and “eraser” ALKBH5 into the cytoplasm where replication and transcription of the viral genome are processed [802]. Since the YTH-domain family 2 (YTHDF2) protein mediates the deadenylation that decays m^6^A-marked transcripts [803,804], replication of the SARS-CoV-2 virus is deemed to be sensitive to the negative regulation by m^6^A “reader” YTHDF2 [802]. An analysis of RNA-seq of 126 COVID-19 patient blood samples from a GEO dataset revealed a strikingly elevated level of m^6^A modification associated with increased expression of CD4^+^ T cells in leukocytes of infected compared to uninfected individuals [805]. A systematic analysis of RNA-seq and clinical data obtained from 100 COVID-19 and 26 non-COVID-19 subjects revealed that the expression of both METTL3 “writers” and FTO “erasers” were increased in the 100 COVID-19 patients but not in non-COVID-19 controls, where m^6^A targeted genes were differentially expressed. However, patients under emergency care exhibited lower m^6^A signature scores compared to controls [806]. The fact that m^6^A modifications in both host and the infecting virus are dynamic interacting events that can be time-sensitive may create additional challenges in the interpretation of the evidence reported. 

An examination of m^6^A modification kinetics in African green monkey kidney epithelial (Vero) cells [807] infected by SARS-CoV-2 discovered distinct peaks during the first 12 h of infection where m^6^A modified SARS-CoV-2 RNA peaked at 10 hpi–12 hpi (~100%+), whereas the relative level of m^6^A modification was significantly higher at 2 hpi (~10%) compared to 4 hpi (~5%) and 6 hpi (4%) [808]. The use of different cell types and the time of extraction post-infection may also conceivably affect the results obtained from infected cells to account for contradictory observations reported in literature. Liu et al. reported that the knockdown of “writers” METTL3/METTL14 and “reader” YTHDF2 increased viral infection/replication, while the knockdown of “eraser” ALKBH5 decreased infection in human hepatocarcinoma (Huh7) cells after 72 hpi [802]. Their results were confirmed by Zannella et al. who used rhein—an inhibitor of m^6^A “erasers”—to knockdown fat mass and obesity-associated protein (FTO) [809] in Vero cells infected by SARS-CoV-2, where a dose-dependent effect was seen after 14 hpi with interference of viral life cycle to complete blockage of infection at the highest dose used [810]. Conversely, works of others have found m^6^A “writers” and “readers” to positively regulate SARS-CoV-2 infection and replication, where their knockdowns decreased viral replication (Table 1, Figure 3).

In human colorectal adeno-carcinoma Caco-2 infected by the SARS-CoV-2 virus, knocking down methyltransferase METTL3 reduced viral load, proviral gene expression and m^6^A levels 24 hpi [251]. Similar results were obtained in Vero cells 24 hpi, where knockdown of METTL3 reduced viral titer, N protein gene expression and copy number, whereas knocking down FTO produced the opposite effect. Interestingly, an increase in METTL3 expression and a decrease in FTO expression were detected at 48 hpi [811]. A reproducible microarray analysis of m^6^A epitranscriptome of peripheral blood samples obtained from severe, mild, and healthy controls not infected by SARS-CoV-2 revealed a significantly higher level of hypermethylated genes that were positively correlated with the methyltransferase RMB15 in severe patients. Knocking down RMB15 in cutaneous T-lymphocyte (HuT 78) cells resulted not only in the decline of total m^6^A methylation levels and expression of genes associated with programmed cell death and inflammatory response, but also rescued lymphocyte apoptosis in vitro at 24 hpi [812]. In stably expressing human ACE2 adenocarcinomic human alveolar basal epithelial (A549^+ACE^) cells, knocking down METTL3 and “readers” YTHDF1/YTHDF3 reduced SARS-CoV-2 infection and subsequent accumulation of proteins by ~3- to 24-fold, in addition to decreased synthesis of the N protein at 48 hpi [813] (Table 1, Figure 3). At this point, it is tempting to speculate regarding the inconsistent results on METTL3 and FTO during SARS-CoV-2 infections.

Intracellular oxidative stress is increased during SARS-CoV-2 infection in a time-dependent manner [296,393]. The knockdown of METTL3 can potentially further exacerbate oxidative stress injury in tested cells [814,815]. The SARS-CoV-2 is a positive-strand virus that promotes viral replication via template switching within the 5′ cap structure [789]. During the early replication cycle, a negative strand uncapped copy of the RNA genome is generated and used as a template to synthesize the 5′ capped positive strand genomes in later stages of viral protein production. Capping activity is enhanced by an oxidative environment but inhibited by the presence of antioxidants [816]. Consequently, exposure to 72 h of the SARS-CoV-2 virus could have significantly elevated oxidative stress and increased viral production. Therefore, the independent replication of results obtained by Liu et al. [802] to further clarify the effects of m^6^A “writers” and “erasers” on SARS-CoV-2 replication is of paramount importance. However, the use of rhein to support the hypothesis that FTO knockdown inhibits SARS-CoV-2 replication may be open to question. There is little doubt that rhein can reversibly bind to FTO and inhibit m^6^A demethylation in vitro [817]; however, rhein is not a selective inhibitor of m^6^A “erasers” that include the ALKB family proteins [818]. Furthermore, rhein is a natural product with an impressive array of pharmacological activities [819], including antiviral mechanisms. Rhein can significantly inhibit IAV adsorption and replication via antioxidant-dependent pathways in vitro [820]. Therefore, if rhein inhibition of IAV is, in part, mediated through the knockdown of FTO, which would increase METTL3, then the results would completely invalidate the findings of Courtney et al., where inhibition of IVA replication in A549 cells was achieved by the knockdown of METTL3 [794] and not by its increase via depletion of FTO “erasers”.

The manipulation of FTO demethylases during the early stages of SARS-CoV-2 infection becomes even more compelling considering the fact that METTL3 knockdown in Huh7 cells can suppress both glycolysis and the mammalian target of rapamycin complex 1 (mTORC1) in patients with hepatocellular carcinoma (HCC) [821], supporting theories that target glucose metabolism and the inhibition of the mTOR pathway as effective treatments for COVID-19 [822,823]. It is also possible that the SARS-CoV-2 virus commandeers host m^6^A modifications to enhance N protein phase separation, promoting viral replication, and hijacking DDX3X via disassembly of SGs. Interestingly, all of these strategies are intricately intertwined with m^6^A modifications, and melatonin is in a most unique position to dismantle the entire viral operational structure using multifaceted maneuvers.

### 7.6. Melatonin Phosphorylation of GSK-3 Increases the m^6^A Demethylase FTO

The SARS-CoV-2 virus hijacks GSK-3 to tune the viscosity and function of condensates formed by its N protein via phosphorylation (Section 6). The hijacking of GSK-3 may also increase m^6^A and associated activities by METTL3 because GSK-3 can phosphorylate m^6^A “eraser” FTO, resulting in lower levels of FTO and higher levels of METTL3 “writers”. In mouse ESCs, knockout of GSK-3 reduced m^6^A nucleotides by 50% compared to wild-type ESCs, reflecting the significant increase in FTO demethylase activities [824,825,826]. Consequently, knockdown of FTO in infected Vero cells not only increased viral titers, but also increased the gene expression and copy number of the N protein [811]. The phosphorylation of FTO by GSK-3β and the subsequent degradation of FTO by ubiquitination downregulates the expression of the transcription factor Myc during myocardial ischemia/reperfusion injury, elevating cardiomyocyte apoptosis and oxidative stress levels [827,828]. Thus, the timely deactivation of GSK-3 by melatonin via inhibition of GSK-3 gene expression and the phosphorylation of GSK-3 can increase FTO levels and reduce METTL3 in the early stages of infection to effectively suppress not only viral phase separation by N protein, but also the global modulation of the epitranscriptome by the SARS-CoV-2 virus (Figure 2).

### 7.7. SARS-CoV-2 Suppresses Innate Immune Responses by Hijacking DDXs to Enhance ALKBH5 and METTL3

The suppression of GSK-3 activities can also prevent the SARS-CoV-2 virus from hijacking DDX3X and disassembling SGs (Section 6.1). RNA helicases of the DEAD-box (DDX) protein family are highly conserved, ATP-dependent enzymes found among most prokaryotic, archaea, eukaryotic, and viral genomes, responsible for critical roles in all aspects of RNA metabolism from transcription and translation to final degradation [829]. DDX proteins assume proviral features during viral infection due to their essential functions in the regulation of cellular stress and survival mechanisms [830]. Many RNA viruses, including hepatitis C virus (HCV) [831], human immunodeficiency virus type I (HIV-1) [832], Japanese encephalitis virus (JEV) [833], severe acute respiratory syndrome coronavirus (SARS-CoV) [834], and even SARS-CoV-2, can bind and hijack host DDX proteins to facilitate and enhance viral genome replication, even though these RNA viruses all express their own RNA helicases [530]. The SARS-CoV-2 N protein hijacking of DDX3X not only enhances viral genome transcription and packing [530], but may also utilize DDX3X to manipulate host antiviral responses via m^6^A “eraser” alpha-ketoglutarate-dependent dioxygenase alkB homolog 5 (ALKBH5) demethylase that can remove specific immune m^6^A modification targets.

ALKBH5 is an m^6^A modification enzyme with the important role of balancing methylation and demethylation of RNAs that modulates RNA metabolism [835]. However, in an in vivo VSV-infection mouse model, ALKBH5 can erase m^6^A-modified antiviral transcripts to prevent IFN translation and inhibit type I IFN production by binding to nuclear DDX46, inducing the retention of antiviral transcripts including MAVS in the nucleus, reducing the expression of MAVS, and inhibiting MAVS-induced activation of IFN-β luciferase reporter [836]. RNA-Seq of severe COVID-19 patient blood samples showed a significant elevation of leukocyte CD4^+^ T cells compared to uninfected controls [805]. ALKBH5 triggers inflammatory cascades by enhancing CD4^+^ T cell response during viral infections. In an experimental autoimmune encephalomyelitis mouse model, ALKBH5 decreased m^6^A modification on interferon-γ (IFN-γ) and C-X-C motif chemokine ligand 2 (CXCL2) mRNA in CD4^+^ T cells, increasing the stability of mRNA transcripts and enhancing corresponding protein expression [837]. Consequently, knockdown of ALKBH5 in Huh 7 cells was found to decrease replication of SARS-CoV-2 72 hpi [802]. DDX3X plays an important role as a direct regulator of ALKBH5, mediating the modulation of demethylation of m^6^As during viral infections.

DDX3X is both an ATPase and an RNA helicase involved in a broad range of RNA metabolic activities including transcription and translation to regulate not only cell cycle progression and apoptosis, but also antiviral and type I IFN immune responses [838,839,840]. As such, DDX3X is a “double-edged sword” during viral replication [532]. In human oral squamous cell carcinoma, the demethylation of mRNAs by ALKBH5 in HEK293T and HeLa cells is mediated by DDX3X [841]. DDX3X is required for direct interaction with ALKBH5 where the ATP domain of DDX3X must interact with the DSBH domain of ALKBH5 in order to stabilize binding between DDX3X and ALKBH5 [842]. DDX3Xs are recruited to SGs during SG assembly [523]. Therefore, by disassembling SGs via manipulation of the GSK-3/Gle1 pathway, the SARS-CoV-2 virus can liberate and sequester DDX3X to facilitate binding with ALKBH5 to enhance immune evasion [802,843], whereas the timely inhibition of the GSK-3/Gle1 pathway during early infection stages by melatonin can protect the assembly of SGs and restrain DDX3Xs from binding excessively with ALKBH5 (Figure 2). 

The SARS-CoV and SARS-CoV-2 viruses manipulate other DEAD-box proteins, including Asp-Glu-Ala-Asp (DEAD)-box polypeptide 5 (DDX5 or p68) to negatively regulate the innate antiviral immune response by increasing METTL3-mediated RNA methylation and the subsequent decay of antiviral transcripts [530,844]. The resulting blockade of the p65 pathway enhances viral replication, whereas the recruitment and interaction between DDX5 and METTL3 can be further exploited and enhanced by viral infections. DDX5^+/-^ mice showed significantly lower levels of viral titers and reduced tissue damage than wild-type controls [845]. Knocking down DDX5 in SARS-CoV significantly hampered the viral replication [834]. While the SARS-CoV-2 spike protein S2 region interacts with the N-terminal of DDX5 to decrease DDX5X binding to METTL3 and increase METTL3 m^6^A modifications. Knocking down DDX5X further intensified the effects of spike protein S2 region elevation of m^6^A modifications in a macrophage lipid uptake model [846]. Interestingly, DDX5 can bind and enhance G3BP1 transcription in an antagonistic, competitive manner with MAGE-B2, where DDX5 binding to G3BP1 is inversely correlated to MAGE-B2 binding [847]. MAGE-B2 represses G3BP mRNA translation by displacing DDX5 helicase from the 5′ UTR [848]. Therefore, spike protein S2 interactions with DDX5 can potentially facilitate and augment N protein disassembly of SGs via inhibition of the SG nucleator G3BP.

### 7.8. G3BP1 Is Repelled by m^6^A METTL3 Modification, but Associates with YTHDF Proteins to Form Stress Granules

During viral infections, SGs assume the responsibility of temporarily sequestering non-translating mRNAs and RNA-binding proteins (RBPs) to stall host bulk translation and limit viral protein accumulation [66,67]. Thus, viruses target the disassembly of SGs via different mechanisms to dismantle innate antiviral immune response [65,849,850,851]. The stress granule protein G3BP1 is strongly repelled by m^6^A in an RNA-sequence-context-dependent manner, which directly and negatively affects the binding of G3BP1 to its targeted sites on mRNAs, with the implication that m^6^A can inhibit G3BP1 binding in certain sequence contexts and that m^6^A can negatively affect the stability of G3BP1 target mRNAs. Knockdown of METTL3, therefore, significantly increased the stability of G3BP1 target mRNAs as a result of increased binding effects, not dissimilar to overexpression of G3BP1 [852,853]. Hence, the regulation of m^6^A modifications and METTL3 expression during the early stages of viral infection may be critical in controlling not only N protein phase separation [854], but also the cascading effects of phase separation and the subsequent disassembly of SGs via interactions with G3BP1 (Section 5.5) (Figure 1 and Figure 2).

G3BP1 is responsible for the nucleation of SGs to promote multiple innate immune antiviral responses [855,856]. m^6^A “readers” YTHDF1, YTHDF2, and YTHDF3 are enriched in SGs under oxidative stress, where YTHDF1/3 clusters around the periphery of G3BP1 proteins while YTHDF2 are colocalized with G3BP1 in SGs to promote SG formation [857]. The activation of YTHDF proteins are inversely correlated with oxidative stress [858], and m^6^A nucleotides interact with YTHDF proteins to lower activation energy barrier and reduce critical size necessary for SG formation [859]. The knockdown of YTHDF1/3, but not YTHDF2, substantially reduced both the size of G3BP1 protein clusters and SG formation in human osteosarcoma (U2OS) cells [857]. Interestingly, knocking down YTHDF1/3 in A549^+ACE2^ reduced SARS-CoV-2 replication and protein synthesis [813]. Conversely, knocking down YTHDF2 had the opposite effect of increasing SARS-CoV-2 replication in Huh7 cells [802] (Table 1). A global analysis of protein–RNA interactions in human lung cancer (Calu-3) cells infected by SARS-CoV-2 revealed that both YTHDF2 and YTHDF3 were slightly upregulated during early stages (8 h) but significantly downregulated in later stages (24 h) [158]. Human ovarian surface epithelial cells (HOSEpiCs) treated with the Ras oncogene exhibited characteristics associated with senescence-associated secretory phenotype (SASP) and reduced levels of YTHDF2 from elevated production of ROS. Treatment with 1 mM melatonin attenuated SASP by upregulation of YTHDF2 and reduction of ROS [860]. Therefore, the timely application of melatonin during early stages of infection may positively regulate YTHDF2 and negatively regulate METTL3 to inhibit viral replication (Figure 2).

### 7.9. Melatonin Modulates the Expression of m^6^A METTL3 Methyltransferase in a Context-Dependent, Pleiotropic Manner

Melatonin elevates the expression of demethylase FTO via suppression of GSK-3 gene expression and the phosphorylation of GSK-3 (Section 7.6). The increase of FTO is naturally associated with a decrease in m^6^A levels. However, an analysis of changes in quantified mRNA expression levels of m^6^A methyltransferase and demethylase in epididymal white adipose tissues obtained from mice subjected to an alimentary obesity model found a significant reduction in the transcription of not only “writers” METTL3, METTL14, but also the “eraser” ALKBH5 in animals treated with 20 mg/kg melatonin (i.p.) for 14 days compared to controls. Conversely, transcriptions for m^6^A “reader” YTHDF2 and “eraser” FTO were markedly increased. Furthermore, m^6^A levels were inhibited by melatonin supplementation in adipocytes examined [861] (Table 2). In vitro work showed that melatonin treatment for 48 h at 1 μmol/L enhanced the stability of mRNAs in extracellular vesicles derived from bone marrow-derived mesenchymal stem cells by reducing transcription of METTL3 to suppress global m^6^A modification levels [862] (Table 2). While long-term cultured ESCs treated with 10 μM melatonin maintained stemness features for over 90 days (45 passages) accompanied by a global decrease in m^6^A modification and a significant reduction of METTL3 in the nuclei of treated ESCs, in addition to changes in expression levels of 2486 genes compared to controls. The reduction in m^6^A modification not only decreased methylation and increased RNA stability of core pluripotency factors Nanog, Sox2, Klf4, and Myc, but also upregulated levels of these transcription factors due to extension of mRNA half-life times [863] (Table 2). Pluripotent stem cells are highly sensitive to subtle changes in the level of oxidative stress in their environment where excess, uncontrolled ROS impact proliferation, differentiation, and genomic stability [864,865]. Oxidative stress and ROS signaling are also critical in the modulation of m^6^A RNA modification.

Cancer cells with aberrant oxidative and antioxidant systems often exhibit dynamic crosstalks between oxidative stress and m^6^A modifications where intracellular ROS levels can change the levels of m^6^A methylation but may also be regulated by m^6^A modifications [866]. An analysis of RNA-seq assays for mouse neuroblastoma (Neuro-2A) cells treated with paraquat (PQ)—an oxidative stress-inducing herbicide—revealed that both oxidative stress as well as antioxidative stress can generate distinct transcriptome distributions of m^6^A peaks that modified circular RNAs (circRNAs) in treated cells, where PQ-treated cells presented abundant m^6^A peaks across the CDS region but exhibited less peaks in the 3′-UTR region; whereas cells pretreated with the antioxidant N-acetylcysteine (NAC) demonstrated the exact opposite effect. PQ treatment increased and decreased m^6^A methylation in 107 and 112 circRNAs, respectively, while NAC pretreatment increased and decreased m^6^A methylation in 156 and 111 circRNAs, respectively [867]. Neuro-2A cells treated with PQ for 3 h caused hypermethylation of total long noncoding RNAs, significantly increasing the expression levels of METTL3 and METTL14 methyltransferases, but decreasing the expression of FTO and ALKBH5 demethylases [868]. Both m^6^A “writers” and “erasers” are sensitive to oxidative stress, and their expression levels can be regulated by oxidative stress. Elevated oxidative stress induced by cobalt chloride (CoCl2) exposure caused a marked downregulation of FTO expression [869]. The expression levels of methyltransferase METTL16 was significantly increased by oxidative stress in both in vitro human nucleus pulposus cells (NPCs) and an intervertebral disc degeneration (IVDD) IVDD animal model in female C57BL/6 mice [870]. Thus, the reduction in m^6^A elevation under oxidative stress conditions by melatonin may be associated with its potent antioxidant features. However, melatonin is a pleiotropic molecule that can also exert an opposite effect to increase METTL3 expression in a high oxidative stress environment.

Chromium (V1) (Cr (VI)) induces oxidative stress via enhanced production of ROS, leading to genomic DNA damage and lipid peroxidation [871]. Male C57BL/6J mice injected with Cr (VI) (16.2 mg/kg i.p. daily × 14) but pretreated with 25 mg/kg (i.p. daily × 14) melatonin all exhibited attenuated cell viability loss, ROS generation, and reduced mitochondrial dynamic imbalance compared to controls. While the in vitro treatment of mouse spermatogonial stem cells (SSCs) with 10 μM Cr (VI) produced a clear loss of m^6^A modification after 1 h, in addition to a marked reduction in METTL3 expression and m^6^A modifications of mitochondrial fusion genes after 4 h of exposure, pretreatment with 50 μM melatonin not only restored METTL3 levels but also attenuated suppression of m^6^A modifications in Cr (VI)-treated ESCs compared to controls [872] (Table 2). Ultimately, melatonin is a “broad-based metabolic buffer” [415] that is used by living organisms in all three domains of life to reduce exogenous and endogenous stress by maintaining redox homeostasis in antioxidant- and prooxidant-dependent and -independent means [873,874,875,876,877,878]. In general, the knockdown of METTL3, METTL14, and ALKBH5 are associated with reduced SARS-CoV-2 viral replication, but knocking down YTHDF2 and FTO has the opposite effect of increasing viral replication during SARS-CoV-2 infection (Section 7.5, Table 1). Consequently, the delicate balance between m^6^A “writers”, “readers”, and “erasers” that directly exert epitranscriptomic and transcriptomic changes may be greatly influenced by elevated oxidative stress and mitochondrial distress produced as a result of viral infection and replication during acute infection and post-infection recovery.

**Table 2:** In vivo and in vitro effects of melatonin on m^6^A modifications by methyltransferases, demethylases, and “readers”.

m^6^A Modification Enzymes	Model/Description	Melatonin Doses	Melatonin’s Effects	Reference
METTL3/METT14	Epididymal WAT/Alimentary obesity mouse model	20 mg/kg IP injection × 14 days	Reduced transcription.	[861]
ALKBH5	Epididymal WAT/Alimentary obesity mouse model	20 mg/kg IP injection × 14 days	Reduced transcription.	[861]
FTO/YTHDF2	Epididymal WAT/Alimentary obesity mouse model	20 mg/kg IP injection × 14 days	Significantly increased transcriptions.	[861]
METTL3	MSC-derived EV/SCI mouse model	1 μmol/L for 48 h.	Reduced transcription	[862]
METTL3	Long-term cultured ESCs	10 μM × 90 days.	Maintained pluripotency of ESCs by significantly reducing METTL3 levels.	[863]
METTL3	Mouse SSC Cr (VI)-induced m^6^A downregulation	50 μM pretreatment	Restored METTL3 levels, attenuated m^6^A modification reduction.	[872]

WAT: white adipose tissue; MSC: mesenchymal stem cell; EV: extracellular vesicle; SCI: spinal cord injury; ESCs: embryonic stem cells; SSC: spermatogonial stem cell; Cr (VI): chromium (VI); (see Abbreviations for additional acronyms).

## 8. Conclusions

As COVID-19 transitions inevitably from pandemic to endemic, it is presently unclear how continued endemic infections from evolving SARS-CoV-2 variants will shape human health in the years to come. The detrimental effects of viral replication and persistence cause excess oxidative stress and mitochondrial distress that not only activate LINE1 derepression and global demethylation resulting in genomic instability, but also induce epitranscriptomic m^6^A RNA modifications that can alter both host and viral RNA methylomes. Consequently, SARS-CoV-2 introduces a complex, fertile landscape that fosters a wide-array of challenging and often unexplained manifestations [879] during acute infection and PASC. The timely application of melatonin as an essential adjuvant during acute infection and post-infection recovery can inhibit viral infection, replication, and persistence to prevent the hijacking of vital host resources and the global modulation of host genes associated with immune evasion and suppression. Future clinical studies on melatonin and SARS-CoV-2 acute infection should examine the different results from variations in dosages as well as timing of supplementation that may present revealing insights on the regulation of viral phase separation by melatonin. Investigations into how melatonin may be used to address multiple symptoms associated with PASC are also of top priority. During evolution, all living organisms have adapted to coexist with viruses with the assistance of melatonin. The SARS-CoV-2 may eventually be well-tolerated by its human host, but perhaps not without the active involvement of melatonin. Further elucidation on the full potential of melatonin in the regulation of epitranscriptomic and transcriptomic modifications by the SARS-CoV-2 virus is, therefore, highly warranted.

## Figures and Tables

**Table 1:** The in vitro effects of m^6^A modifications by methyltransferases METTL3/METTL14, “readers” YTHDF1/2/3, and demethylases ALKHB5/FTO on SARS-CoV-2 viral replication and protein expression.

m^6^A Modification Enzymes	Cell Line/Extraction Time	Method	Effects	Reference
METTL3	Caco-2/24 hpi	Knockdown	Decreased replication	[251]
METTL3/METTL14	Huh7/72 hpi	Knockdown	Increased replication	[802]
YTHDF2	Huh7/72 hpi	Knockdown	Increased replication	[802]
ALKHB5	Huh7/72 hpi	Knockdown	Decreased replication	[802]
FTO	Vero/14 hpi	Knockdown	Demethylase inhibitor dose-dependent interference with viral lifecyle with complete blockage of infection at highest dose.	[810]
METTL3	Vero/24 hpi	Knockdown	Decreased viral titer, N protein copy number and expression	[811]
METTL3	Vero/24 hpi	Overexpression	Elevated m^6^A modification	[811]
FTO	Vero/24 hpi	Knockdown	Increased viral titer, N protein copy number and expression	[811]
RBM15	HuT 78/24 hpi	Knockdown	Inhibited inflammatory gene expression, lymphocyte apoptosis	[812]
METTL3	A549^+ACE/^48 hpi	Knockdown	Reduced replication, synthesis of viral RNA and N protein	[813]
YTHDF1/YTHDF3	A549^+ACE^/48 hpi	Knockdown	Reduced replication, synthesis of viral RNA and N protein	[813]

hpi: hour post-infection.

## Data Availability

Not applicable.

## Abbreviations

4-HNE	4-hydroxynonenal
ALKBH5	alpha-ketoglutarate-dependent dioxygenase alkB homolog 5
Ca^2+^	calcium
CL	cardiolipin
CNS	central nervous system
DNA	deoxyribonucleic acid
DMR	differentially methylated region
EBOV	Ebola virus
ER	endoplasmic reticulum
FTO	fat mass and obesity-associated protein
GSK	glycogen synthase kinase
HPI	hour post-infection
IB	inclusion body
IBM	inner boundary membrane
IDR	intrinsically disordered region
IFN	interferon
IMM	inner mitochondrial membrane
I.P.	intraperitoneal
ISG	interferon-stimulated gene
ISR	integrated stress response
JAK-STAT	Janus kinase-signal transducers and activators of transcription
K^+^	potassium ion
LINE1, L1	long interspersed nuclear element 1
m^6^A	*N^6^*-methyladenosine
METTL3	methyltransferase 3
METTL14	methyltransferase 14
mPTP	mitochondrial permeability transition pore
mRNA	messenger RNA
NLRP3	NLR pyrin domain containing 3
Nrf2	nuclear factor erythroid 2-related factor
Nsp1	nonstructural protein 1
PASC	post-acute sequelae of COVID-19
PBMC	peripheral blood mononuclear cells
PI	post-infection
RdRp	RNA-dependent RNA polymerase
RBP	RNA-binding protein
RIRR	ROS-induced ROS release
RNA	ribonucleic acid
RNA-seq	RNA sequencing
RNP	ribonucleoprotein
ROS	reactive oxygen species
RSV	respiratory syncytial virus
RT	reverse transcriptase
RTE	retrotransposable element, retrotransposon
SG	stress granule
S/R	serine/arginine
TE	transposable element
VSV	vesicular stomatitis virus
YTHDF2	YTH-domain family 2
ZIKV	Zika virus
